# Observable Classification Patterns and Diagnostic Uncertainty in Parotid Ultrasound: A Multimethod Secondary Analysis

**DOI:** 10.3390/diagnostics16172793

**Published:** 2026-08-30

**Authors:** Lukas Pillong, Oliver Walzer, Marlene Peters, Ida Ohnesorg, Jan Palm, Victoria Bozzato, Malvina Garner, Bernhard Schick, Alessandro Bozzato, Thomas Jäger, Adrian Müller, Susan Pulham, Manfred Voges

**Affiliations:** 1 Department of Otorhinolaryngology, Saarland University, Kirrberger Straße 100, 66421 Homburg, Germany; oliver.walzer@uks.eu (O.W.); victoria.bozzato@uks.eu (V.B.); bernhard.schick@uks.eu (B.S.); alessandro.bozzato@uks.eu (A.B.); 2 Medical Faculty, Saarland University, Kirrberger Straße 100, 66421 Homburg, Germany; mape00008@uni-saarland.de (M.P.); thomas.jaeger@uni-saarland.de (T.J.); 3 Department of Internal Medicine I—Hematology, Oncology, Clinical Immunology and Rheumatology, Saarland University Medical Center, Kirrberger Straße 100, 66421 Homburg, Germany; ida.ohnesorg@uks.eu; 4 Department of Radiation Oncology, Saarland University Medical Center, Kirrberger Straße 100, 66421 Homburg, Germany; jan.palm@uks.eu; 5 Department of Neuroradiology, Saarland University Medical Center, Kirrberger Straße 100, 66421 Homburg, Germany; malvina.garner@uks.eu; 6 Department of Computer Science and Microsystems Technology, University of Applied Sciences Kaiserslautern, Amerikastraße 1, 66482 Zweibrücken, Germany; adrian.mueller@hs-kl.de; 7 Faculty of Business Administration and Economics, Saarland University of Applied Sciences (Hochschule für Technik und Wirtschaft, htw saar), Waldhausweg 14, 66123 Saarbrücken, Germany; susan.pulham@htwsaar.de; 8 Department of Radiology, Saarland University Medical Center, Kirrberger Straße 100, 66421 Homburg, Germany; manfred.voges@uks.eu

**Keywords:** parotid gland, ultrasound, diagnostic uncertainty, interobserver variability, decision trees, signal detection theory, clinical decision-making

## Abstract

**Background/Objectives:** Parotid ultrasound requires integration of sonographic morphology with clinical context under uncertainty, but conventional agreement and performance metrics provide limited insight into examiner-dependent classification patterns. Building on previously derived examiner-specific surrogate models, we applied a behavioral decision-science framework to examine observable classification patterns and case-level ambiguity. **Methods:** In this exploratory retrospective single-center secondary analysis, six examiners rated predefined ultrasound image sets from 149 histopathologically verified lesions. Surrogate-tree structure, feature–reference-class associations, signal detection metrics, and case-level ambiguity were analyzed. Internal robustness was assessed using cross-validation and sensitivity analyses. **Results:** Pruned surrogate trees identified a context-based classification pattern, a morphology-based classification pattern, and a hierarchical morphology-context pattern. Tumor history and lesion boundary were the only retained upper-level classification cues and showed the strongest overall categorical and sonographic associations with the histopathological reference class, respectively. Across 30 cross-validation trees, boundary was the root split in 22 and tumor history in eight; no other root feature occurred. Examiners differed in diagnostic discrimination and estimated decision criterion, with Examiner 3 showing the highest discrimination. Boundary remained the sonographic feature most strongly associated with the histopathological reference class across sensitivity analyses. Descriptor disagreement was associated with decision disagreement and error burden, and four cases showed unanimous but incorrect classifications. **Conclusions:** In this exploratory retrospective single-center cohort of histopathologically verified lesions, examiner-dependent classification was characterized by observable, model-derived cue structure, feature-use/reference-association alignment, estimated decision-criterion patterns, and case-level ambiguity. These cohort-specific findings do not directly measure examiner cognition and should not be generalized to unselected parotid-ultrasound populations; however, the complementary analyses provide an empirically grounded framework for characterizing observable examiner-dependent classification and identifying testable targets for prospective studies of examiner feedback, descriptor standardization, and decision support.

## 1. Introduction

Ultrasound is a central first-line imaging modality for assessing parotid gland lesions [1,2]. In clinical practice, sonographic evaluation contributes to the initial differentiation between lesions that appear likely benign and those that raise suspicion for malignancy. This assessment integrates morphological descriptors, including lesion boundary, contour, echogenicity, texture, size, vascularization, acoustic features, and number of tumors, with clinical context variables [3,4,5,6]. However, benign and malignant parotid lesions may show overlapping sonographic appearances [2], and individual descriptors can be difficult to categorize consistently. Prior work on multiparametric parotid imaging has emphasized both the diagnostic relevance of sonographic morphology and the overlap between benign and malignant lesion patterns [2,3,7].

Interobserver variability remains an important challenge in parotid ultrasound assessment [8,9]. Structured approaches, including ultrasound parotid imaging reporting and data systems, have been proposed to improve categorization and reproducibility, and previous studies have specifically evaluated interobserver agreement in parotid lesion assessment [10]. Across diagnostic imaging more generally, interobserver variability studies commonly quantify agreement using statistical measures, although a recent methodological systematic review identified substantial heterogeneity in study conduct, reporting, and interpretation [11].

In previous work using the same examiner dataset, we reported diagnostic performance, interobserver agreement, and examiner-specific surrogate decision trees that approximated individual benign/malignant labeling patterns [9]. That study focused on observer performance, agreement, and the descriptive structure of the surrogate trees, showing that examiner-dependent classification patterns can be represented explicitly using structured sonographic descriptors and clinical metadata [9]. These surrogate trees, however, should not be interpreted as direct representations of cognitive processes. Rather, they provide model-derived approximations of observable classification behavior and may therefore provide the starting point for the complementary analyses conducted in the present study.

Medical image interpretation involves both perception and decision-making under uncertainty [12,13,14]. Benign/malignant classification draws on multiple clinical and imaging-derived cues [7,9]. From a behavioral decision-science perspective, observable classification patterns can be discussed in relation to diagnostic cues, model-derived classification structure, feature–reference-class associations, and threshold-based concepts of decision-making [15,16,17]. Simplified decision strategies are not necessarily erroneous; heuristics can be efficient and adaptive when they rely on information valid within the decision environment [18,19,20]. In medical decision-making, intuitive or heuristic processes should therefore not automatically be equated with diagnostic error, and dual-process accounts require a more nuanced interpretation than a simple opposition between intuition and analysis [18,19,20].

Signal detection theory and observer-performance methodology provide additional tools for characterizing examiner-dependent classification patterns [21,22,23,24]. In benign/malignant classification, diagnostic discrimination must be distinguished from the decision criterion used to assign a malignant classification. Threshold-based approaches to clinical decision-making have long emphasized that diagnostic actions depend on disease probability, test properties, and the relative consequences of false-positive and false-negative classifications [17,25]. Diagnostic uncertainty is likewise difficult to capture using a single metric because it may include subjective, dynamic, and decision-relevant components [13].

The present exploratory secondary analysis examined what additional insights into examiner-dependent benign/malignant classification could be obtained from examiner-specific surrogate-tree outputs and associated rating data within a behavioral decision-science framework. Specifically, the present study added (1) analysis of the alignment between model-retained features and their associations with the histopathological reference class, (2) examiner-specific diagnostic discrimination and estimated decision criterion, and (3) case-level relationships among descriptor disagreement, classification disagreement, and diagnostic error, including misleading-consensus cases. The analysis concerned observable, model-derived patterns rather than direct measures of cognition and was intended to add an interpretative dimension to our previous decision-tree analysis [9], thereby providing a conceptual foundation for future prospective studies of diagnostic decision-making under uncertainty.

## 2. Materials and Methods

### 2.1. Study Design and Analytical Framework

In this study, 149 parotid lesion cases with available histopathological reference diagnoses were independently evaluated by six examiners. For each case, examiners assigned a binary benign/malignant classification based on predefined sonographic descriptors, including boundary, contour, echogenicity, texture, size, vascularization, acoustic features, and number of tumors, together with available clinical information such as age, gender, tumor history, B symptoms, facial nerve palsy, alcohol consumption, and nicotine consumption.

**Figure 1:**
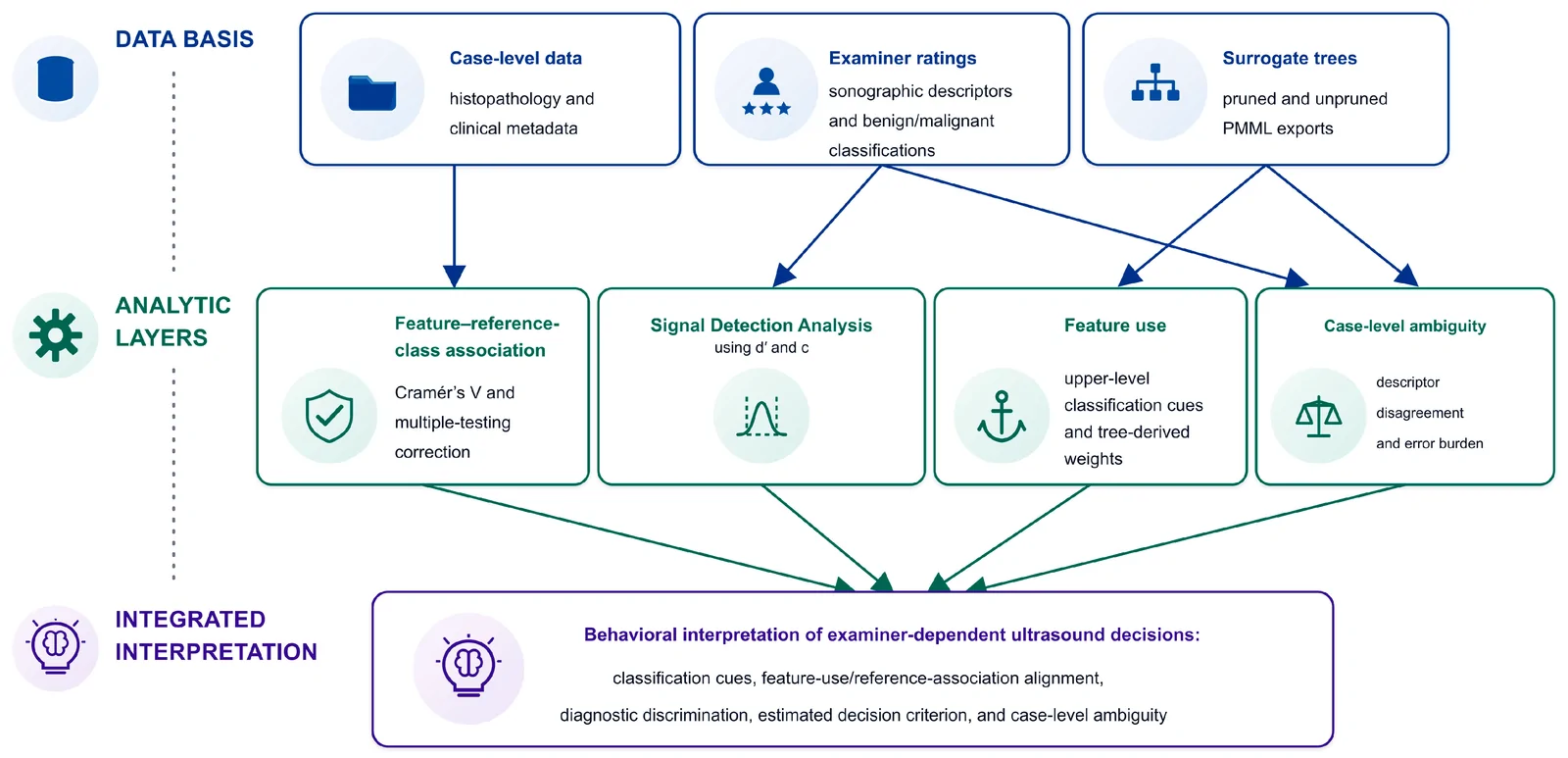
Behavioral decision-science analysis framework. The figure summarizes the analytical framework used to reinterpret examiner-dependent parotid ultrasound assessment from a behavioral decision-science perspective. Case-level data, examiner ratings, and examiner-specific surrogate trees were integrated across four analytic layers: feature–reference-class association, signal detection, tree-derived feature use, and observable case-level ambiguity. These layers were used to characterize examiner-dependent classification patterns in terms of model-derived classification cues, feature-use/reference-association alignment, diagnostic discrimination and decision criterion, observable case-level ambiguity, and misleading-consensus cases.

The overall analytical framework is shown in Figure 1.

### 2.2. Patient Cohort, Image Material, and Examiner Rating Procedure

The present analysis was based on a retrospective single-center cohort of patients with parotid gland lesions and an available histopathological reference diagnosis. Cases were included if evaluable ultrasound image material and the corresponding histopathological reference diagnosis were available.

Ultrasound examinations had been acquired in routine clinical settings using high-resolution ultrasound. Image material was reviewed for evaluability before examiner rating. Cases were considered evaluable if the lesion was sufficiently visible for morphological assessment and if image quality allowed assessment of predefined sonographic descriptors, including lesion boundary, contour, echogenicity, texture, size, vascularization, acoustic features, and number of tumors.

Six examiners from different specialties and levels of experience independently evaluated predefined ultrasound image sets. The panel comprised three otorhinolaryngologists—Examiner 1 (DEGUM Level III, >20 years’ experience in head and neck ultrasound), Examiner 2 (DEGUM Level II, 19 years’ experience in head and neck ultrasound), and Examiner 3 (6 years’ experience in head and neck ultrasound)—as well as a radiation oncologist (Examiner 4, >20 years’ experience in head and neck imaging), a final-year medical student (Examiner 5, 1 year of experience in head and neck imaging), and a neuroradiologist (Examiner 6, 7 years’ experience in head and neck imaging), as reported previously [9]. The examiner panel was treated as a heterogeneous group of individual examiners rather than as representatives of specific medical specialties. Accordingly, examiner-level analyses were intended to characterize observable individual classification patterns, not to compare specialties or professional groups.

All examiners received a standardized evaluation template containing predefined sonographic descriptors and available clinical metadata. Clinical context variables included age, gender, B symptoms, facial nerve palsy, alcohol consumption, nicotine consumption, and tumor history. Tumor history was defined as any documented previous neoplastic disease and was provided as a binary structured context variable (yes/no); it was not restricted to primary parotid tumors and did not encode tumor type, anatomical site, or timing. These variables were provided as structured input variables because ultrasound interpretation in routine clinical practice is commonly contextualized by clinical information.

Examiners rated cases independently and were blinded to the histopathological reference diagnosis and to the ratings of the other examiners. No feedback on diagnostic performance, model-derived cues, or classification variability was provided before or during the rating procedure.

### 2.3. Dataset and Examiner Ratings

The expected number of examiner-case ratings was 894, corresponding to 149 cases assessed by six examiners. A total of 892 ratings were available. Examiners 3 and 6 each contributed 148 rather than 149 ratings because one examiner-case assessment was unavailable for each examiner. The specific reasons for these two isolated missing assessments were not documented, and the missingness mechanism could therefore not be determined. Accordingly, no specific missingness mechanism was assumed and no ratings were imputed.

Missing examiner-case ratings were excluded from analyses requiring the respective examiner-case observation. Examiner-level diagnostic performance and signal detection metrics were calculated using available ratings for each examiner. Case-level decision disagreement, descriptor disagreement, and error burden were calculated using the available examiner ratings for each case. For feature-reference-class association analyses, sonographic descriptor ratings were aggregated to the case level using the majority rule described below; cases without an evaluable aggregated descriptor for a given feature were excluded from the corresponding feature-specific association analysis.

For the analyses, data were structured at three complementary levels: the examiner-rating level, the case level, and the tree/split-node level. Examiner-rating-level data were used for diagnostic performance and signal detection analyses. Case-level data were used for feature-reference-class association and ambiguity analyses. Tree/split-node-level data were derived from examiner-specific surrogate decision trees and used for tree-derived feature-use analyses.

Examiner specialty and experience were documented but were not used as grouping variables because, with only six examiners, experience, specialty, and training environment were closely intertwined and could not be analyzed as independent explanatory factors. Examiners were designated as Examiner 1–6 throughout the analysis, and results were interpreted at the level of individual examiner-specific classification patterns.

### 2.4. Histopathological Reference Standard

For all primary binary analyses, histopathological findings were dichotomized into malignant versus benign reference classes. Cases labeled as malignant in the histopathological reference were assigned to the malignant class. All other histopathological entities, including Warthin tumor, pleomorphic adenoma, sialadenitis, cysts, lymph nodes, angioma, and other benign or non-malignant findings, were assigned to the benign class.

Each examiner’s binary benign/malignant classification was compared with this dichotomized histopathological reference standard.

### 2.5. KNIME Workflow and Examiner-Specific Surrogate Trees

Examiner-specific surrogate decision trees were generated to approximate the observable benign/malignant classification patterns of each examiner. For each examiner, the examiner-assigned binary benign/malignant classification served as the outcome variable. Consistent with the previously published surrogate-modeling framework [9], the candidate predictor set comprised tumor history, facial nerve palsy, nicotine consumption, boundary, contour, echogenicity, texture, vascularization, acoustic features, and number of tumors. Age, gender, B symptoms, alcohol consumption, and lesion size were not included as candidate predictors during tree induction. The present secondary analysis retained this established predictor set without re-specification and therefore did not re-evaluate the incremental contribution of lesion size to tree induction.

These surrogate trees were used as model-based approximations of observable examiner-specific classification patterns. They were not interpreted as direct representations of cognitive processes, but as structured outputs that allowed exploratory analysis of which variables were most relevant for approximating each examiner’s classifications. In the present analysis, the term ‘model-derived classification cue’ refers to a feature retained in an examiner-specific surrogate decision tree that contributed to approximating the examiner’s observable benign/malignant labeling pattern. Upper-level classification cues were defined as features retained in pruned trees, particularly root splits or early split variables. This terminology is descriptive and model-derived; it does not imply that the corresponding feature was consciously used by the examiner or that it represents a directly measured cognitive process. Accordingly, throughout this study, the term ‘tree-derived feature use’ denotes variable retention and structural prominence within a surrogate tree rather than directly observed examiner-level information use. The decision-tree workflow was implemented in KNIME Analytics Platform version 5.8.2 (KNIME AG, Zurich, Switzerland). The dataset was imported using an Excel Reader node. Examiner-specific subsets were generated using Rule-based Row Filter nodes. Subsequent preprocessing and variable selection were performed using Rule Engine and Column Filter nodes. For each examiner, decision trees were trained using the Decision Tree Learner node. Decision trees were generated using gain ratio as the split criterion. Pruned trees were obtained using minimum description length (MDL) pruning. A minimum of five cases per node was required. Model visualization was performed within KNIME using Decision Tree View nodes.

Tree models were exported from the Decision Tree Learner output in Predictive Model Markup Language (PMML) format using PMML Writer nodes. The exported PMML files were subsequently parsed and analyzed outside KNIME using custom Python scripts. The KNIME workflow used for tree generation and PMML export is provided as Supplementary Figure S4.

Both pruned and unpruned decision trees were analyzed. Pruned trees were used to characterize the dominant upper-level classification structure of each examiner-specific surrogate model, with stability evaluated separately through fold-specific cross-validation. Unpruned trees were used only for exploratory characterization of downstream tree-derived feature-use patterns and sample-specific refinements beyond the retained upper-level classification cues; they were not interpreted as fixed examiner-specific decision rules.

For each tree, the following structural characteristics were extracted from the PMML files: root split, internal split nodes, terminal leaves, total nodes, maximum depth, split features, node depth, and record count at each split node. Detailed structural characteristics of the pruned and unpruned surrogate trees are provided in Supplementary Table S2.

### 2.6. Tree-Derived Weighted Feature-Use Score

To quantify tree-derived feature use in the examiner-specific surrogate trees, a weighted feature-use score was calculated for each feature, examiner, and tree type. The score was designed to give greater weight to features appearing earlier in the tree and at split nodes containing a larger number of cases.

For each internal split node, the contribution of the corresponding split feature was calculated as:

Weighted split contribution = (record count at split node/total number of cases in the tree) × [1/(depth + 1)]

Feature-specific contributions were summed across all split nodes using the same feature. The resulting weighted scores were normalized within each tree to obtain the relative feature-use share. The weighting specification was researcher-defined and represented one plausible operationalization rather than a unique or validated measure of feature use.

Features were grouped into two broad categories. Clinical context variables included tumor history, facial nerve palsy, and nicotine consumption. Sonographic morphology variables included boundary, contour, echogenicity, texture, vascularization, acoustic features, and number of tumors. Lesion size was not part of the tree-derived feature-use scoring but was retained in the separate feature–reference-class association and descriptor-disagreement analyses.

Pruned trees were used to identify robust upper-level classification cues. Unpruned trees were used to characterize examiner-specific downstream tree-derived feature-use patterns.

### 2.7. Feature–Reference-Class Associations

Feature–reference-class association was defined as the strength of the univariable association between each clinical or sonographic feature and the binary histopathological reference class.

Clinical categorical variables were analyzed on the case level. For sonographic descriptors, examiner ratings were aggregated to the case level using majority aggregation for each descriptor. A descriptor was considered evaluable for a given case if the most frequently assigned category accounted for more than 50% of available examiner ratings. The resulting majority-aggregated descriptor was treated as an operational case-level summary of the examiner ratings for association with the case-level histopathological reference class, not as a direct measurement of the underlying lesion characteristic or as evidence of examiner consensus. Individual ratings, including minority ratings, were retained for the separate entropy-based descriptor-disagreement analyses.

Associations between categorical features and the binary histopathological reference class were quantified using Cramér’s V as an effect-size measure. Higher Cramér’s V values indicate stronger univariable associations with the histopathological reference class within the present cohort. Statistical significance was assessed using chi-square tests or Fisher’s exact tests where appropriate. For the main majority-aggregation analysis, the 14 categorical feature–reference-class association tests reported in Supplementary Table S10, comprising six clinical variables and eight sonographic descriptors, were treated as a single test family and adjusted using the Benjamini–Hochberg false-discovery-rate procedure. In the aggregation-rule sensitivity analyses, the adjustment was recalculated separately across the same 14-feature family within each aggregation rule. Interpretation emphasized effect sizes and uncertainty, whereas unadjusted and FDR-adjusted *p*-values were treated as secondary inferential descriptors.

Age was examined separately as a continuous cohort characteristic using the point-biserial correlation with the binary histopathological reference class, coded as benign = 0 and malignant = 1. A percentile 95% confidence interval was calculated using 5000 case-level bootstrap resamples. Because Cramér’s V is defined for categorical variables, age was retained in its continuous form rather than categorized using an arbitrary cutoff and was therefore reported separately from the categorical Cramér’s V ranking. The feature-use/reference-association alignment analysis was restricted to variables included as candidate predictors in the surrogate-tree workflow; because age was not included in tree induction, no tree-derived feature-use component was available for age.

The majority-aggregation approach represented the main feature–reference-class association analysis.

### 2.8. Alignment Between Tree-Derived Feature Use and Feature–Reference-Class Association

To evaluate the alignment between tree-derived feature use and feature–reference-class association, examiner-specific feature-use scores were compared with the corresponding Cramér’s V estimates.

For each tree, an exploratory weighted feature-use/reference-association alignment index was calculated as:

Weighted feature-use/reference-association alignment index = Σ(relative feature-use share*_i_* × Cramér’s V*_i_*)

where *_i_* denotes the features retained in the tree. This descriptive index summarizes the extent to which tree-derived feature use emphasized features showing stronger univariable associations with the histopathological reference class in the present cohort.

The researcher-defined exploratory weighted feature-use/reference-association alignment index was calculated separately for pruned and unpruned trees. Pruned trees were used to assess the alignment between robust upper-level classification cues and their reference-class associations, whereas unpruned trees were used to assess the corresponding alignment of downstream tree-derived feature-use patterns.

### 2.9. Signal Detection Analysis

Signal detection analysis was used to separate examiner-specific diagnostic discrimination from the decision criterion (c) for malignant classification. In the present study, c was interpreted as a within-cohort estimate of the observed operating point rather than as a stable psychological trait or a directly measured clinical action threshold.

For each examiner, a 2 × 2 contingency table was constructed using the examiner’s binary benign/malignant classification and the binary histopathological reference standard. True positives (TP), false positives (FP), false negatives (FN), and true negatives (TN) were used to calculate accuracy, sensitivity, specificity, positive predictive value, and negative predictive value.

The hit rate was defined as sensitivity:

Hit rate = TP/(TP + FN)

The false alarm rate was defined as:

False alarm rate = FP/(FP + TN)

Diagnostic discrimination was calculated as:

d′ = z(hit rate) − z(false alarm rate)

The decision criterion was calculated as:

c = −0.5 × [z(hit rate) + z(false alarm rate)]

where z denotes the inverse cumulative distribution function of the standard normal distribution.

Higher d′ values indicate better discrimination between benign and malignant cases. Positive values of c indicate a relatively more conservative observed operating point for malignant classification, whereas negative values indicate a relatively more liberal operating point. These estimates describe response tendencies within the present case set and may vary with case mix, task instructions, and clinical context.

For bootstrap resamples in which the hit rate or false alarm rate was 0 or 1, a log-linear correction was applied to avoid infinite z values. Corrected rates were calculated as:

Corrected hit rate = (TP + 0.5)/(TP + FN + 1)

Corrected false alarm rate = (FP + 0.5)/(FP + TN + 1)

Confidence intervals for examiner-level performance and signal detection metrics were obtained using bootstrap resampling as described below.

### 2.10. Observable Case-Level Ambiguity Analysis

Observable case-level ambiguity was analyzed to characterize the extent to which individual cases elicited disagreement in sonographic descriptor assessment and final benign/malignant classification. In the present study, case-level ambiguity was operationalized through inter-examiner disagreement and error burden rather than through subjective uncertainty ratings.

For each case, decision disagreement was calculated as the normalized binary entropy of benign/malignant classifications across examiners. Decision disagreement was 0 when all available examiners assigned the same classification and maximal when benign and malignant classifications were equally distributed.

For each sonographic descriptor, descriptor disagreement was calculated as normalized categorical entropy across available examiner ratings. The overall descriptor disagreement score for each case was defined as the mean normalized entropy across the eight predefined sonographic descriptors and served as an observable measure of inter-examiner variability in feature categorization. Definitions and computational details for the normalized entropy measures are provided in Supplementary Tables S4 and S5.

For each case, error burden was calculated as:

Error burden = number of incorrect examiner classifications/number of available examiner classifications

Cases were descriptively categorized according to their observable classification patterns as correct consensus cases, partial disagreement cases, maximal disagreement cases, or misleading-consensus cases. Correct consensus cases were defined as cases in which examiners agreed and were correct. Maximal disagreement cases corresponded to a 3:3 split in benign/malignant classifications. Misleading-consensus cases were defined as cases in which all available examiners assigned the same benign/malignant classification, but this consensus classification was discordant with the histopathological reference standard.

Associations between descriptor disagreement, decision disagreement, and error burden were assessed using Spearman rank correlation. Before rank transformation, normalized entropy values were rounded to 12 decimal places so that algebraically identical values were treated as ties; two-sided *p*-values were calculated using the standard t approximation with n − 2 degrees of freedom. To assess robustness to tied ranks, two-sided permutation *p*-values were additionally calculated for the three central correlations reported with bootstrap confidence intervals using 100,000 case-label permutations, average ranks, and NumPy’s Generator with the PCG64 bit generator and seed 42. Monte Carlo *p*-values were calculated as (b + 1)/(B + 1), where b denotes the number of permuted absolute correlations at least as large as the observed absolute correlation and B = 100,000.

### 2.11. Robustness and Sensitivity Analyses

Four additional robustness and sensitivity analyses were performed.

For each examiner, the X-Partitioner node in KNIME was configured with five validation folds, stratified sampling according to the examiner-assigned benign/malignant classification, and a fixed random seed. Within each fold, a pruned examiner-specific decision tree was trained using the same tree-induction settings as in the full-data analysis, including gain-ratio splitting, minimum description length (MDL) pruning, and a minimum of five cases per node. Fold-specific trees were exported in PMML format and parsed using custom Python scripts. For each fold-specific tree, the root split, second-level split features, all split features, number of internal nodes, number of leaves, total number of nodes, maximum depth, and training-set record count were extracted. Root-split stability was summarized as the frequency with which each feature was retained as the root-level classification cue across the five folds for each examiner.

Second, examiner-level diagnostic performance and signal detection metrics were accompanied by 95% confidence intervals obtained by case-level bootstrap resampling within each examiner. In total, 5000 bootstrap samples were generated per examiner using resampling with replacement. Accuracy, sensitivity, specificity, positive predictive value, negative predictive value, d′, and c were recalculated for each bootstrap sample. Percentile-based 95% confidence intervals were calculated from the resulting bootstrap distributions. Computationally, case-level resampling was implemented by multinomial resampling of the examiner-specific TP, FP, FN, and TN counts, which is equivalent for metrics determined solely by the 2 × 2 table. Random numbers were generated with NumPy’s Generator using the PCG64 bit generator and seed 42. Metric values with a zero denominator were treated as missing for that bootstrap sample, and the 95% confidence limits were the 2.5th and 97.5th percentiles calculated using linear interpolation.

Third, the robustness of feature–reference-class association findings to the aggregation of sonographic descriptor ratings was assessed using stricter case-level aggregation rules. The main analysis used majority aggregation, defined as the top descriptor category representing more than 50% of available examiner ratings. Sensitivity analyses required the top descriptor category to be supported by at least four examiner ratings, which differs from the >50% rule only when fewer than six ratings were available, or by at least five examiner ratings. For each aggregation approach, associations between categorical features and the binary histopathological reference class were recalculated using Cramér’s V, and multiple-testing correction was repeated using the false discovery rate.

Fourth, the dependence of the weighted feature-use/reference-association alignment index on the researcher-defined weighting scheme was examined using four specifications. The original W0 scheme, node support × 1/(depth + 1), was compared with W1, unweighted split frequency; W2, node support without depth weighting; and W3, node support × 2^(−depth)^. Contributions were normalized within each tree before relative feature-use shares and weighted alignment indices were recalculated. Comparisons of index values and rankings across weighting schemes were descriptive and were not subjected to significance testing.

### 2.12. Statistical Analysis and Software

Analyses were performed at the corresponding data level. Examiner performance and signal detection metrics were calculated at the examiner-rating level. Feature–reference-class associations and observable case-level ambiguity metrics were calculated at the case level. Tree-derived feature-use scores were calculated at the split-node level and summarized by examiner and tree type. Cross-validation-based stability analyses were performed on fold-specific pruned tree models.

The KNIME workflow was used for examiner-specific surrogate-tree generation and PMML export. The original PMML parsing, post-processing, statistical analyses, table generation, and figure generation were performed using custom scripts in Python 3.13.5 (Python Software Foundation, Wilmington, DE, USA), with pandas 2.2.3, NumPy 2.3.5, SciPy 1.17.0, and Matplotlib 3.10.8. PMML files were parsed as XML files to extract tree topology, split variables, node depth, record counts, terminal node information, and fold-specific root splits. The secondary weighting, aggregation, examiner-specific association, and central-effect bootstrap analyses were run in Python 3.12.13 with NumPy 2.3.5, pandas 2.2.3, openpyxl 3.1.5, python-docx 1.2.0, and Python’s xml.etree.ElementTree parser. Statistical functions for these secondary analyses were independently implemented and validated against the corresponding primary point estimates. The case bootstrap used NumPy Generator/PCG64 with seed 42 and 5000 resamples.

Categorical associations with the histopathological reference class were summarized using Cramér’s V and tested using chi-square or Fisher’s exact tests as appropriate. Multiple-testing correction was performed using the false discovery rate. Correlations between ambiguity metrics were assessed using Spearman rank correlation. Bootstrap confidence intervals were calculated using percentile intervals from case-level resampling. Statistical analyses were interpreted as exploratory and hypothesis-generating, given the secondary-analysis design, the limited number of examiners, and the model-based nature of the surrogate decision-tree interpretation.

## 3. Results

### 3.1. Analytical Framework and Dataset Structure

A total of 149 parotid lesion cases with an available histopathological reference diagnosis were included in the analysis. Six examiners independently assessed predefined sonographic descriptors and available clinical information and assigned a binary benign/malignant classification. Across all cases, 892 of 894 expected examiner-case ratings were available for analysis. Examiners 3 and 6 each contributed 148 ratings, whereas the remaining examiners contributed 149 ratings.

The histopathological reference class was benign in 102 cases (68.5%) and malignant in 47 cases (31.5%). These proportions describe the histopathologically verified study cohort and should not be interpreted as representative disease prevalence in an unselected population undergoing parotid ultrasound. Detailed cohort, histopathology, and rating-completeness information is provided in Supplementary Table S1. Tumor history was positive in 35/149 cases (23.5%): 10/102 benign lesions (9.8%) and 25/47 malignant lesions (53.2%).

Age was higher among malignant than benign cases (median [IQR], 75.0 [59.0–83.0] vs. 61.0 [53.0–67.0] years). The point-biserial correlation between age and the malignant reference class was 0.314 (95% bootstrap CI, 0.155–0.463). This cohort-specific univariable association was reported separately and was not incorporated into the categorical feature ranking or the surrogate-tree analyses.

The analysis integrated three complementary data levels: case-level clinical and histopathological data, examiner-level sonographic ratings and benign/malignant classifications, and examiner-specific pruned and unpruned surrogate decision trees exported in Predictive Model Markup Language format from the KNIME workflow.

Four complementary analytic layers were considered: feature–reference-class associations, tree-derived feature use, signal detection analysis, and observable case-level ambiguity.

Feature–reference-class association analysis quantified the univariable associations between clinical or sonographic features and the binary histopathological reference class. Tree-derived feature use was used to characterize robust upper-level classification cues and examiner-specific downstream tree-derived feature-use patterns. Signal detection analysis separated diagnostic discrimination from decision criterion. Observable case-level ambiguity analysis examined how inter-examiner disagreement in sonographic descriptor assessment was associated with disagreement in final benign/malignant classification and with per-case error burden.

### 3.2. Pruned Surrogate Trees Show Three Upper-Level Classification Patterns

**Figure 2:**
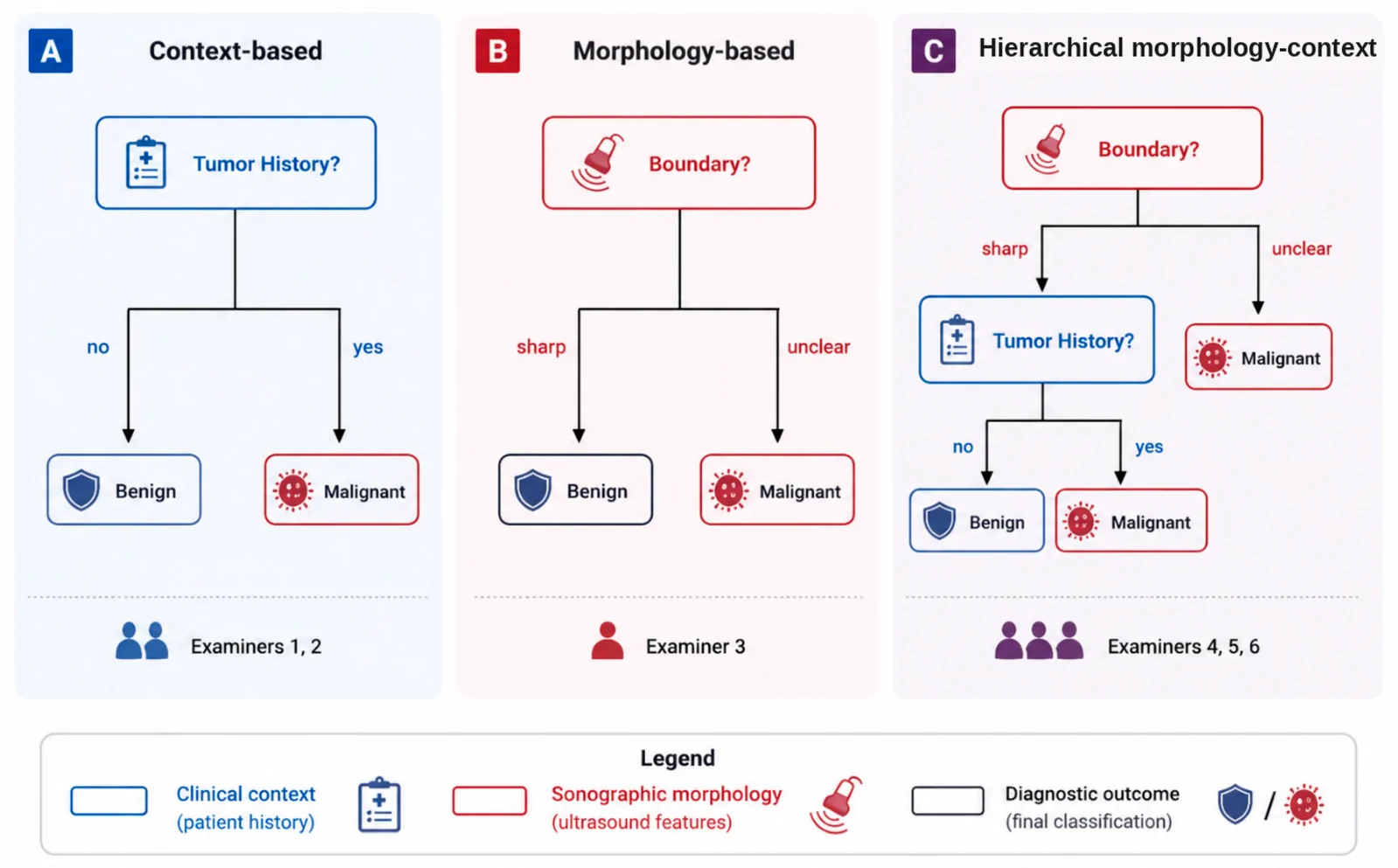
Pruned surrogate-tree typology of examiner-specific classification patterns. Pruned examiner-specific surrogate trees showed three upper-level classification patterns. (**Panel A**) shows a context-based classification pattern, in which tumor history served as the sole retained upper-level classification cue; this pattern was observed in Examiners 1 and 2. (**Panel B**) shows a morphology-based classification pattern, in which boundary served as the sole retained upper-level classification cue; this pattern was observed in Examiner 3. (**Panel C**) shows a hierarchical morphology-context pattern, in which boundary formed the root split and tumor history provided second-level refinement for cases with sharp lesion boundaries; this pattern was observed in Examiners 4, 5, and 6. Clinical context variables are shown in blue, and sonographic morphology variables are shown in red.

Pruned examiner-specific surrogate trees showed three upper-level classification patterns (Table 1, Figure 2).

Examiners 1 and 2 were best approximated by a context-based classification pattern in which tumor history represented the sole retained upper-level classification cue. Examiner 3 was best approximated by a morphology-based classification pattern in which boundary represented the sole retained upper-level classification cue. Examiners 4, 5, and 6 were approximated by a hierarchical morphology-context pattern, in which boundary formed the root split and tumor history provided second-level refinement for cases with sharp lesion boundaries.

After pruning, the examiner-specific surrogate trees retained two central upper-level classification cues: tumor history and boundary. These variables characterized the upper-level classification structure of the pruned surrogate trees. Additional clinical or sonographic variables appeared in the unpruned trees and were analyzed as downstream tree-derived feature-use patterns.

The resulting pruned-tree typology showed that clinical-context and sonographic-morphology variables occupied different structural positions across examiner-specific surrogate models.

In a stratified 5-fold cross-validation sensitivity analysis of the pruned examiner-specific surrogate trees, the retained root splits were restricted to the same two upper-level classification cues identified in the full-data analysis. Across all 30 fold-specific pruned trees, boundary was retained as the root-level classification cue in 22 models and tumor history in 8 models. No other feature was retained at the root level. These frequencies describe the root-level predictive structure of the fold-specific surrogate models and do not, consciously or unconsciously, establish the order in which examiners considered the available features. Examiner 3 showed complete stability of the morphology-based pattern, with boundary retained as the root split in all five folds. Examiners 5 and 6 also showed boundary as the root split in all five folds. Examiners 1 and 2 were predominantly context-based but showed partial root-split variability, with boundary emerging as the root split in two and one of five folds, respectively. Examiner-level summaries and detailed fold-specific results are provided in Supplementary Tables S7A and S7B, respectively, while root-split frequencies are visualized in Supplementary Figure S5. At the level of fold-specific classification patterns, the full-data classification pattern was the most frequent pattern for Examiners 1–4 and 6, whereas Examiner 5 showed a morphology-based pattern in three of five folds.

Thus, cross-validation supported the restriction of the root-level feature set to boundary and tumor history and the relative predominance of the full-data classification patterns, but it did not establish stability of the complete examiner-specific tree topologies.

### 3.3. Signal Detection Analysis Separates Diagnostic Discrimination from Estimated Decision Criterion

**Figure 3:**
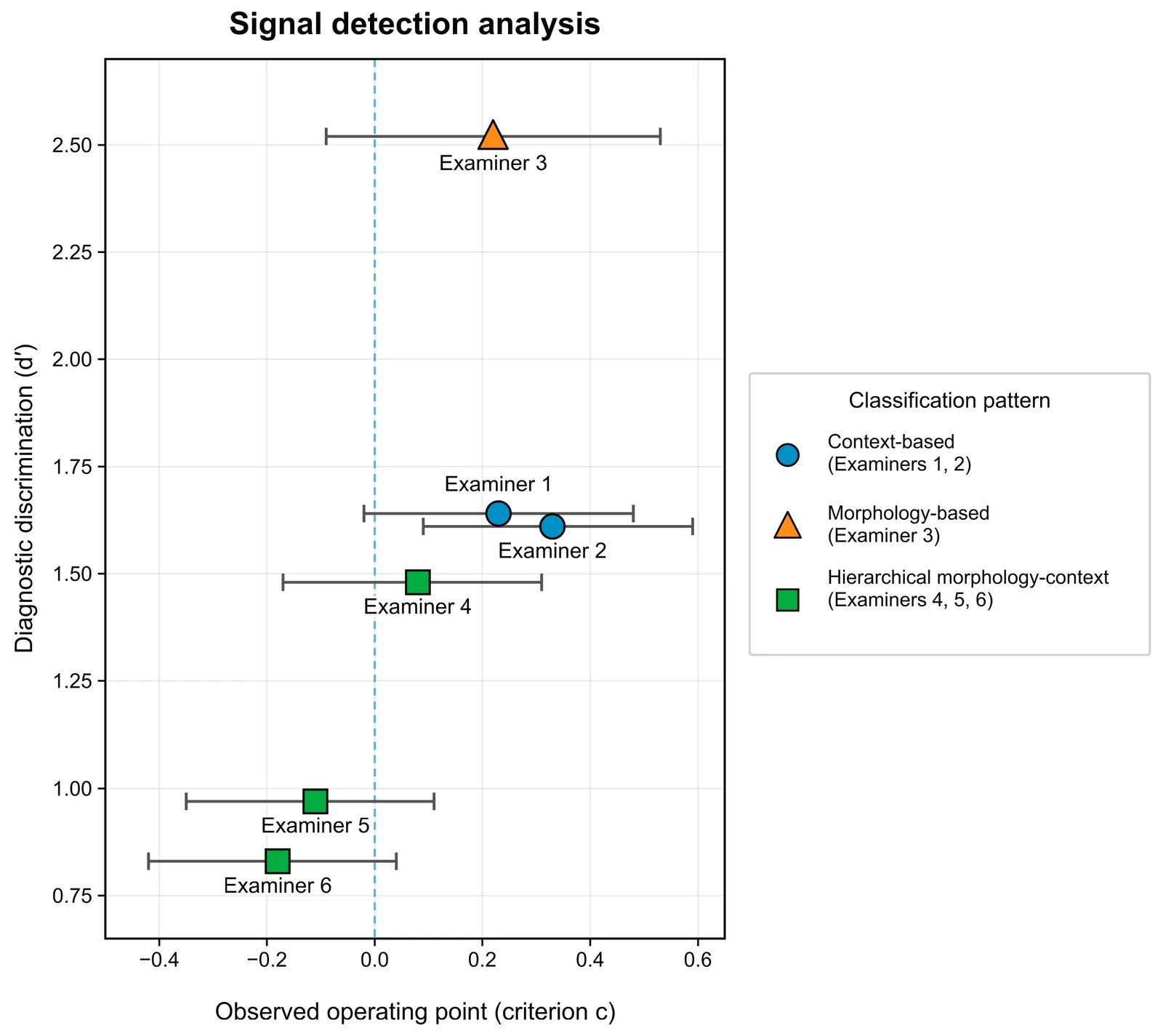
Signal detection analysis of examiner-level benign/malignant classification. The scatter plot shows diagnostic discrimination (d′) on the y-axis and decision criterion (c) on the x-axis for each examiner. Higher d′ values indicate better discrimination between benign and malignant cases. Positive c values indicate relatively more conservative observed operating points for malignant classification, whereas negative c values indicate relatively more liberal observed operating points. Marker shapes and labels indicate the pruned surrogate-tree classification pattern. Horizontal error bars indicate case-bootstrap 95% confidence intervals for c; numerical confidence intervals for both d′ and c are provided in Supplementary Table S8.

Diagnostic performance varied across examiners (Table 1), and signal detection analysis decomposed this variation into diagnostic discrimination and estimated decision criterion (Figure 3).

Examiner 3 showed the highest diagnostic discrimination, with d′ = 2.52, accompanied by a mildly positive criterion estimate. Examiners 1 and 2 showed comparable discrimination values, with d′ = 1.64 and d′ = 1.61, respectively, and positive decision criteria for malignant classification.

Examiners 5 and 6 showed lower discrimination values, with d′ = 0.97 and d′ = 0.83, respectively. Both also showed negative criterion estimates, corresponding to more liberal observed operating points for malignant classification within the present cohort. This pattern was accompanied by lower specificity values in these examiners. Examiner 4 showed an intermediate profile, with d′ = 1.48 and a near-neutral criterion estimate.

Bootstrap confidence intervals supported the overall examiner-level pattern of signal detection metrics. Examiner 3 showed the highest diagnostic discrimination, with d′ = 2.52 [95% CI, 2.01–3.27]. Examiners 5 and 6 showed lower discrimination values, with d′ = 0.97 [95% CI, 0.54–1.47] and d′ = 0.83 [95% CI, 0.39–1.34], respectively. Decision criterion estimates were positive for Examiners 1–4 and negative for Examiners 5 and 6, although confidence intervals for c overlapped zero in several examiners. Full bootstrap confidence intervals for diagnostic performance and signal detection metrics are provided in Supplementary Table S8.

Thus, examiner-level performance differed in diagnostic discrimination and in the estimated within-cohort operating point for malignant classification.

### 3.4. Retained Upper-Level Classification Cues Show Strong Reference-Class Associations

**Figure 4:**
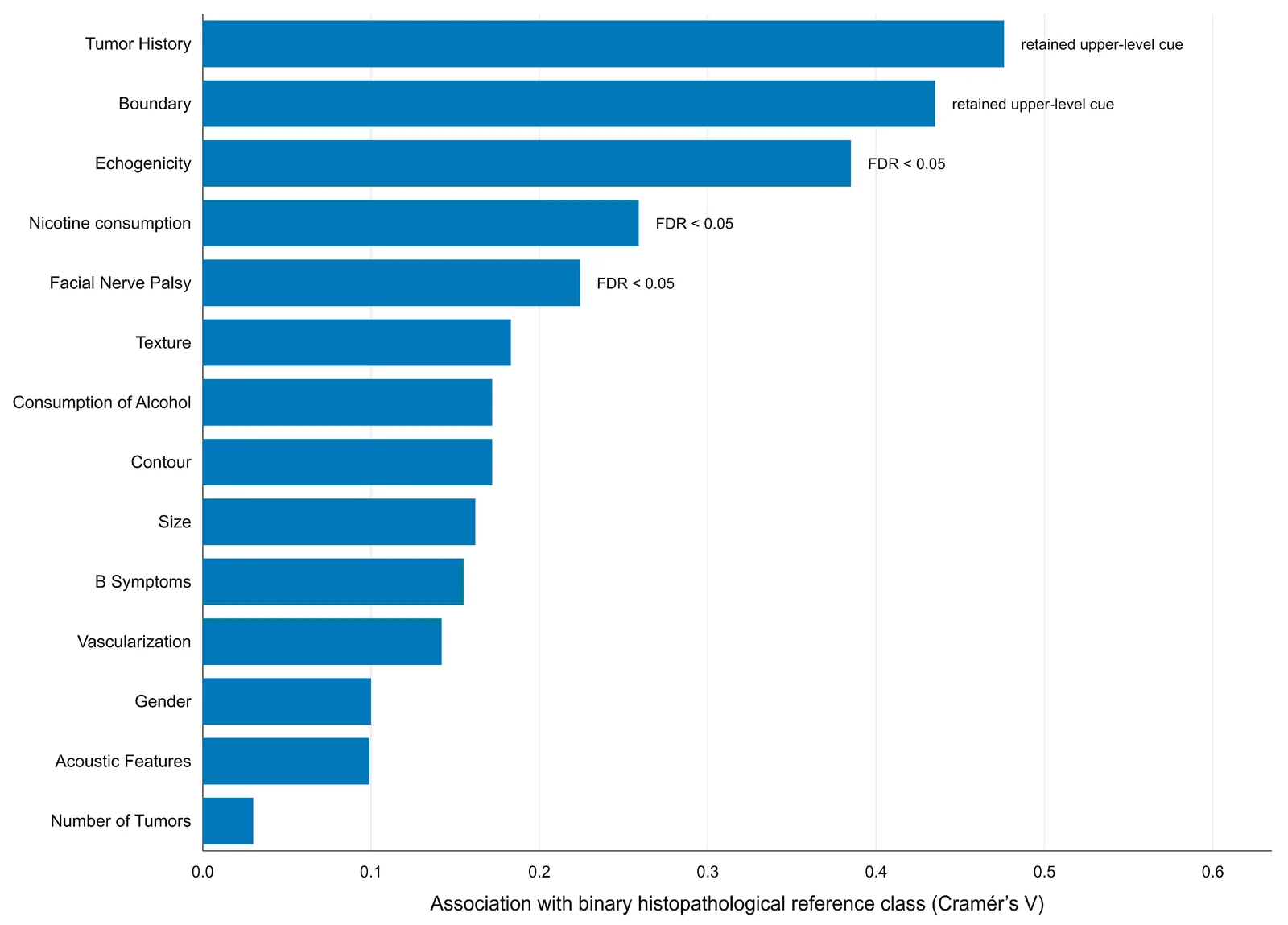
Feature–reference-class associations of clinical and sonographic features. The bar plot shows the strength of the univariable association between categorical clinical or sonographic features and the binary histopathological reference class. Effect sizes are reported as Cramér’s V, with higher values indicating stronger association with the reference class. Tumor history showed the strongest association overall, and boundary was the sonographic descriptor most strongly associated with the histopathological reference class. Associations for tumor history and boundary remained statistically significant after FDR correction and are annotated by their retained upper-level-cue status; the remaining FDR-significant features are marked “FDR < 0.05”. Exact *p* values and evaluable case counts are provided in Supplementary Table S10.

Associations between clinical and sonographic features and the binary histopathological reference class were quantified using Cramér’s V (Figure 4; Supplementary Table S10).

Among categorical variables, tumor history showed the strongest association with the histopathological reference class, with Cramér’s V = 0.476 (95% CI, 0.320–0.628). Boundary was the strongest sonographic descriptor, with Cramér’s V = 0.435 (95% CI, 0.274–0.588). Echogenicity showed the next-largest sonographic point estimate, with Cramér’s V = 0.385.

After correction for multiple testing using the false discovery rate (FDR), associations for tumor history, boundary, echogenicity, nicotine consumption, and facial nerve palsy remained statistically significant. The two features retained as robust upper-level classification cues in the pruned surrogate trees, tumor history and boundary, also showed the two strongest categorical associations with the histopathological reference class.

The main majority rule and the requirement of at least four concordant ratings produced nearly identical results, as expected for cases with six available ratings. Under the high-consensus requirement of at least five concordant ratings, the number of cases evaluable for boundary decreased from 144 to 127, while its Cramér’s V increased from 0.435 to 0.484 and boundary showed the strongest overall categorical association. By contrast, the association for echogenicity decreased from 0.385 to 0.292 and was no longer significant after FDR correction. Thus, robustness was strongest for the relative prominence of boundary, whereas estimates for other sonographic descriptors were more sensitive to the aggregation rule. Detailed results are provided in Supplementary Table S9 and Supplementary Figure S6. In a complementary examiner-specific sensitivity analysis, boundary was also the highest-ranked sonographic descriptor for each of the six examiners (Supplementary Table S12).

The upper-level classification cues retained in the pruned surrogate trees corresponded to features showing the strongest univariable associations with the histopathological reference class in the present cohort.

### 3.5. Tree-Derived Feature-Use Patterns in Unpruned Trees Show Examiner-Specific Downstream Refinement

While the pruned trees identified robust upper-level classification cues, the unpruned surrogate trees showed examiner-specific downstream tree-derived feature-use patterns. The unpruned trees were dominated by tumor history in Examiners 1 and 2 and by boundary in Examiners 3–6, with different downstream refinements involving facial nerve palsy, nicotine consumption, echogenicity, contour, acoustic features, vascularization, and number of tumors. Exploratory weighted feature-use/reference-association alignment indices were higher when the trees emphasized tumor history and boundary and lower when downstream refinement involved features with weaker univariable associations with the histopathological reference class (Supplementary Figure S1; Supplementary Tables S3 and S6). Weighting sensitivity differed substantially between pruned and unpruned trees. Across the pruned trees, the largest change in the weighted feature-use/reference-association alignment index relative to the original W0 specification was 2.47%. Across the unpruned trees, the maximum change reached 51.60%, mainly under W1, which assigned equal weight to all split nodes. Thus, alignment estimates based on the upper-level pruned structures were comparatively stable, whereas indices and feature rankings derived from the deeper exploratory structures depended materially on the researcher-defined weighting scheme (Supplementary Table S11).

These analyses support a distinction between robust upper-level classification cues retained in pruned trees and examiner-specific downstream tree-derived feature-use patterns observed in unpruned trees.

### 3.6. Observable Case-Level Ambiguity: Associations Among Descriptor Disagreement, Decision Disagreement, and Error Burden

**Figure 5:**
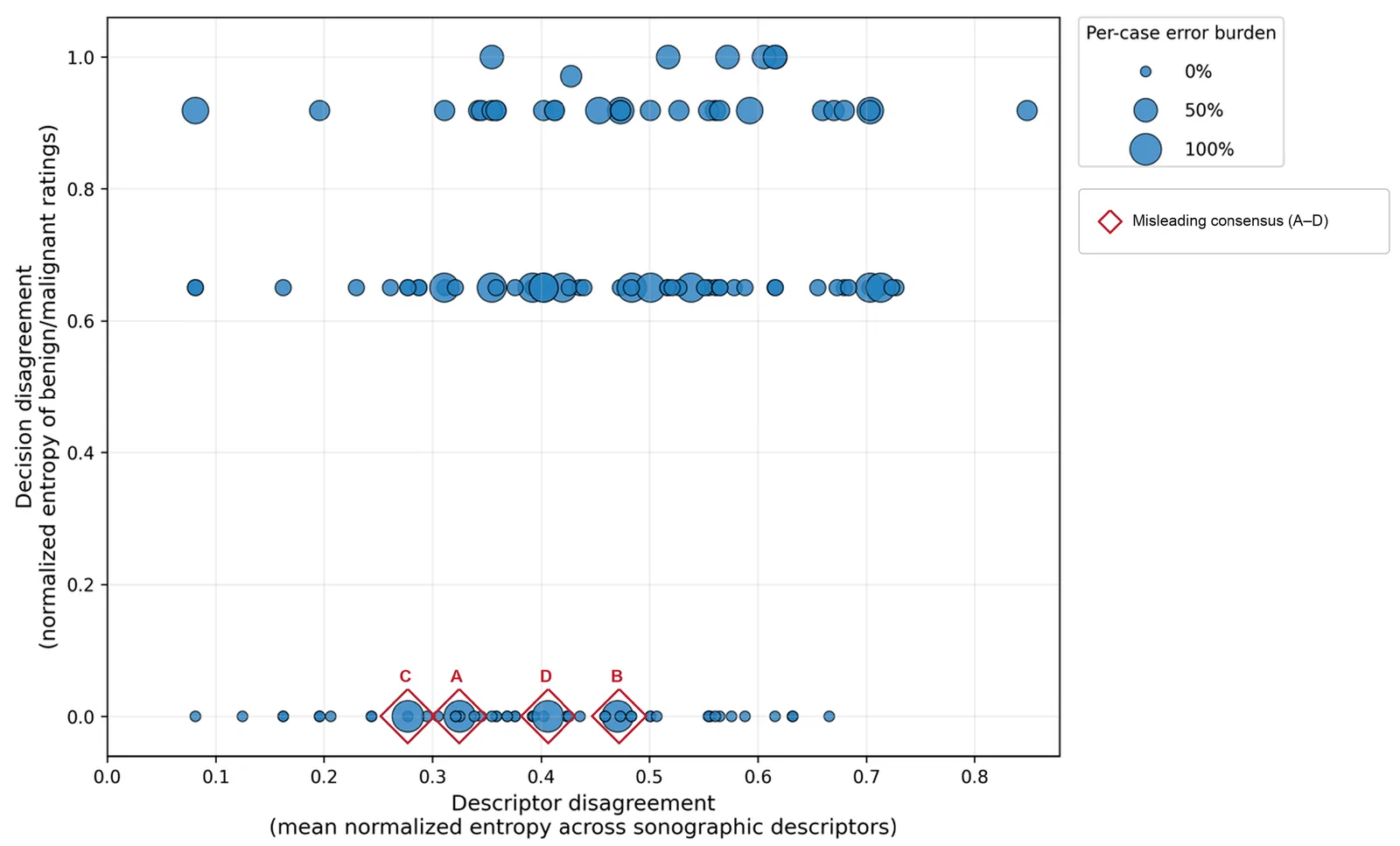
Observable case-level ambiguity in parotid ultrasound assessment. Each point represents one case. The x-axis shows descriptor disagreement, defined as the mean normalized entropy across sonographic descriptors. The y-axis shows decision disagreement, defined as the normalized entropy of benign/malignant classifications across examiners. Marker size reflects per-case error burden, calculated as the proportion of incorrect examiner classifications for each case. Descriptor disagreement was associated with decision disagreement (Spearman ρ = 0.251, 95% CI, 0.097–0.392) and with per-case error burden (Spearman ρ = 0.179, 95% CI, 0.022–0.323). Cases with high decision disagreement represent cases with pronounced inter-examiner disagreement, whereas cases with high error burden but low decision disagreement represent potential misleading-consensus patterns. The four misleading-consensus cases are highlighted by open red diamonds and labeled A–D, corresponding to panels A–D in Supplementary Figure S7 (A, case 49; B, case 69; C, case 120; D, case 127).

Observable case-level ambiguity was examined at the level of individual cases using descriptor disagreement, decision disagreement, and per-case error burden (Figure 5, Supplementary Table S4).

Overall descriptor disagreement was positively but modestly associated with decision disagreement (Spearman ρ = 0.251, 95% CI, 0.097–0.392; *p* = 0.002) and per-case error burden (ρ = 0.179, 95% CI, 0.022–0.323; *p* = 0.029).

Boundary disagreement showed the strongest descriptor-specific association with decision disagreement (Spearman ρ = 0.361, 95% CI, 0.206–0.500; Supplementary Figure S2 and Supplementary Tables S5 and S13). This finding corresponded to the role of boundary as both the sonographic feature most strongly associated with the histopathological reference class and a retained upper-level classification cue in four of the six full-data pruned surrogate trees.

Permutation-based sensitivity analyses produced concordant results (permutation *p* < 0.001 for boundary disagreement versus decision disagreement, *p* = 0.002 for overall descriptor disagreement versus decision disagreement, and *p* = 0.028 for overall descriptor disagreement versus error burden; Supplementary Table S13).

The distribution of case-level decision patterns is shown in Supplementary Figure S3. Fifty-seven cases showed correct consensus across examiners, whereas four were misleading-consensus cases, in which all available examiners assigned the same benign/malignant classification, but this classification was discordant with the histopathological reference standard. Six cases showed maximal decision disagreement, corresponding to a 3:3 split in benign/malignant classifications.

A high per-case error burden occurred both in cases with pronounced inter-examiner disagreement and in cases with examiner consensus.

### 3.7. Misleading-Consensus Cases

Four cases met the predefined criteria for misleading-consensus cases. These included one histopathologically malignant case classified as benign by all available examiners and three histopathologically benign cases classified as malignant by all available examiners. The benign misleading-consensus cases included one sialadenitis and two pleomorphic adenomas.

These cases were characterized by an absence of decision disagreement despite maximal error burden. In contrast, maximal disagreement cases were characterized by an equal distribution of benign and malignant classifications across examiners. This distinction differentiated cases with observable inter-examiner disagreement from cases characterized by complete examiner consensus despite discordance with the histopathological reference standard.

Descriptive post hoc inspection showed different cue configurations across these misleading-consensus cases. The malignant case, classified as benign by all examiners, was consistently described as sharply circumscribed and homogeneous, with additional benign-leaning features, including a round contour, hypoechogenicity, and posterior acoustic enhancement. In contrast, the benign cases classified as malignant showed suspicious morphological or clinical features. The inflammatory lesion with sialadenitis and foreign-body granulomas was characterized by unclear boundary, diffuse vascularization, inhomogeneous texture, tumor history, and facial nerve palsy. The two pleomorphic adenomas classified as malignant showed combinations of unclear boundary, lobulated or irregular contour, larger lesion size, and, in one case, facial nerve palsy. Representative ultrasound images of these four cases are provided in Supplementary Figure S7.

## 4. Discussion

### 4.1. Principal Findings

In this exploratory secondary analysis, we used a behavioral decision-science framework to characterize observable examiner-dependent benign/malignant classification of predefined parotid ultrasound image sets across four complementary analytical levels: model-derived upper-level classification structure, alignment between model-retained feature prominence and feature–reference-class associations, signal-detection estimates, and observable case-level ambiguity. The pruned surrogate trees yielded a context-based classification pattern, a morphology-based classification pattern, and a hierarchical morphology-context pattern. Tumor history and lesion boundary were the dominant upper-level classification cues and showed the strongest overall categorical and sonographic associations, respectively, with the histopathological reference class. Examiner differences were also apparent in diagnostic discrimination and estimated decision criterion, whereas descriptor disagreement was associated with decision disagreement and per-case error burden.

Across the sonographic analyses, boundary showed the clearest convergence: it had the strongest sonographic reference-class association, was a recurrent root-level feature in the pruned surrogate models, and was the descriptor whose disagreement was most strongly associated with disagreement in final classification.

Robustness analyses supported the relative stability of tumor history and boundary as root-level features, while indicating greater uncertainty in second-level tree structure, decision-criterion estimates, and associations for descriptors other than boundary. These analyses extend conventional interobserver assessment, which commonly focuses on aggregate agreement metrics, predominantly intraclass correlation coefficients (ICCs) and kappa statistics [8].

### 4.2. Simplified Model-Derived Cue Structures in Relation to Ecological Rationality

The pruned surrogate trees approximated observable examiner-specific classification patterns using a small number of dominant upper-level classification cues, predominantly tumor history and lesion boundary. We interpret these retained variables as model-level predictors of the final classification labels; whether and how the corresponding features were used during clinical reasoning remains a hypothesis for prospective process-tracing studies. At a conceptual level, these sparse model-derived structures can nevertheless be considered in relation to cue-based accounts of judgment and decision-making under uncertainty [15,16,18].

At the model-structure level, this concentration on a limited number of cues is compatible with concepts of ecological rationality and non-compensatory cue-based simplification. Heuristics may be efficient and adaptive when they fit the structure of the decision environment [18,26,27], whereas the medical decision-making literature cautions against equating intuitive reasoning with diagnostic error [19,20,27].

The prominence of a dominant upper-level cue within the surrogate models resembles the sparse structure described by the One-Clever-Cue concept and related fast-and-frugal approaches [18,26,28]. However, the present data do not establish that examiners sequentially searched for cues, terminated their information search after identifying a discriminating cue, or applied the Take-the-Best heuristic during classification [28].

### 4.3. Alignment Between Model-Retained Features and Feature–Reference-Class Associations

A central finding was the alignment between features retained by the surrogate models and their univariable associations with the histopathological reference class. In the primary feature–reference-class association analysis, tumor history showed the strongest categorical association with the histopathological reference class, whereas boundary showed the strongest association among the sonographic descriptors. Both features were also retained as robust upper-level classification cues in the full-data pruned surrogate trees.

The prominence of tumor history is clinically plausible: a documented history of a relevant previous malignancy may raise suspicion of metastatic involvement of intraparotid lymph nodes, particularly after cutaneous squamous cell carcinoma or malignant melanoma of the head and neck [29,30,31]. However, the binary variable did not distinguish the type, anatomical site, or timing of the previous neoplastic disease and should therefore be interpreted as a broad clinical-context marker rather than as a disease-specific predictor.

Descriptor-aggregation sensitivity analyses supported the relative prominence of boundary across all three aggregation approaches. Under the strictest high-consensus rule, boundary showed the strongest overall categorical association with the histopathological reference class; however, this threshold reduced the number of evaluable cases and preferentially retained lesions with more consistent descriptor ratings. The higher boundary effect estimate should therefore not be interpreted as evidence that stricter aggregation more accurately captures the underlying lesion characteristic, because it may partly reflect the exclusion of cases with less consistent descriptor ratings. The more robust finding was the relative prominence of boundary across aggregation rules, whereas the magnitude and ranking of other descriptor associations were more aggregation-dependent (Supplementary Table S9 and Supplementary Figure S6).

The unpruned trees incorporated additional downstream variables, but these patterns require more cautious interpretation. Although the heuristics literature suggests that experience may influence reliance on highly valid information [18,26], experience, specialty, and training environment were closely intertwined in the present panel. In addition, our previous analysis found no consistent association between surrogate-tree complexity and diagnostic performance [9]; whether experience contributed to the observed model-derived feature-use patterns therefore remains unresolved. Weighting sensitivity further supported the distinction between tree levels: pruned-tree feature-use/reference-association alignment indices changed by no more than 2.47%, whereas unpruned-tree indices changed by up to 51.60%. Alignment between upper-level feature prominence and reference-class association was therefore comparatively stable, whereas quantitative interpretations of deeper structures were materially weighting-dependent (Supplementary Table S11).

### 4.4. Decision Criterion and Signal Detection

Signal detection analysis provided a complementary analytical layer by separating diagnostic discrimination from decision criterion. This distinction is well established in diagnostic imaging and observer-performance research [12,14,24]. Metz emphasized that ROC methodology distinguishes between the inherent diagnostic capacity of image-based interpretation and tendencies to under-read or over-read [23,24]. More broadly, observer-performance methodology treats the human observer as an integral component of the diagnostic imaging process [12,14,24].

Examiner 3 showed the highest diagnostic discrimination, whereas Examiners 5 and 6 showed lower discrimination together with more liberal observed operating points for malignant classification. This distinction illustrates why differences in specificity may reflect different combinations of discrimination and response tendency rather than a unitary difference in diagnostic ability. These examiner-specific observations were descriptive and cannot be attributed to differences in specialty, training, or experience in the present six-examiner panel.

Threshold models provide a conceptual framework for understanding why diagnostic operating points may vary with disease probability, task conditions, test properties, and the relative consequences of classification errors [17,25]. However, these factors were not manipulated, and assessments were not repeated across contexts. In addition, confidence intervals for c included zero for several examiners. The criterion estimates should therefore be interpreted as within-cohort estimates of observed operating points rather than as stable psychological thresholds, enduring examiner traits, or evidence of specific clinical preferences.

### 4.5. Boundary as an Informative Cue and a Locus of Variability

Boundary showed a convergent pattern across three analytically distinct levels: it had the strongest sonographic association with the histopathological reference class (Cramér’s V = 0.435), was retained as the root-level classification cue in four of the six full-data pruned surrogate trees, and showed the strongest descriptor-specific association between disagreement in feature assessment and disagreement in final classification (ρ = 0.361). Although these convergent findings support boundary as a candidate for prospective investigation, they do not establish that reducing disagreement in boundary assessment would improve diagnostic accuracy.

This pattern is consistent with earlier ultrasound studies reporting well-defined boundaries more frequently in benign lesions and ill-defined boundaries more frequently in malignant lesions [4,5,32]. More recent reviews similarly describe benign parotid tumors as more often regular and well-defined and malignant lesions as more often irregular or heterogeneous; however, they also emphasize substantial overlap between benign and malignant sonographic appearances, particularly because pleomorphic adenomas may appear irregular or heterogeneous [2,7,33].

A systematic review of parotid basal cell adenocarcinoma likewise described the difficulty of preoperative differentiation from benign basal cell adenoma and highlighted the complementary role of clinical assessment [34].

These findings illustrate the distinction between feature–reference-class association and descriptor-rating consistency: boundary may show a comparatively strong association with the reference class at the cohort level while its categorization remains variable across examiners in individual cases.

### 4.6. Case-Level Ambiguity and Misleading Consensus

Observable case-level ambiguity varied across cases. Higher descriptor disagreement was associated with greater decision disagreement and higher per-case error burden; however, the modest effect sizes indicate that descriptor disagreement is one measurable correlate of classification variability and error rather than a comprehensive explanation. The entropy-based measures characterize observable case-level ambiguity rather than clinicians’ subjective diagnostic uncertainty, which is a subjective and dynamic construct [13]. Among the sonographic descriptors, boundary disagreement showed the strongest association with final classification disagreement, identifying a testable correlate of classification variability.

Misleading-consensus cases showed a different pattern: all examiners assigned the same binary classification, but the unanimous classification was discordant with the histopathological reference class. Post hoc review suggested that these classifications were supported by coherent clinical and sonographic cue configurations rather than by isolated or arbitrary findings. In these cases, however, the prevailing configuration resembled the opposite reference class, with malignant lesions displaying predominantly benign-leaning features and benign lesions displaying concordant malignancy-associated features. Given that only four cases met this definition and that their inspection was post hoc and descriptive, the observed cue configurations are presented as illustrative, hypothesis-generating examples rather than as a reproducible phenotype of misleading consensus.

This pattern is compatible with the lens model distinction between observer judgment and the environmental criterion [35,36]. Because cue–criterion relationships are probabilistic, a feature may be informative at the cohort level without uniquely identifying the disease state in every case [36]. Several generally informative cues may therefore combine to support a plausible but incorrect classification [36]. The present analysis cannot distinguish whether this reflects limited predictability, unavailable discriminating information, or nonlinear relationships among features [36]. Nevertheless, these cases illustrate that complete examiner agreement should not automatically be equated with diagnostic correctness.

### 4.7. Directions for Prospective Research and Development

Because no training, feedback, or decision-support intervention was evaluated, the present findings identify targets for prospective research rather than established educational or clinical applications. Future studies could test descriptor-focused training, feedback informed by diagnostic discrimination and observed operating points, and the use of ambiguity or misleading-consensus cases for examiner calibration. Decision-support studies could also assess whether presenting information on descriptor disagreement, relevant cue configurations, or warnings about potentially unreliable consensus adds value beyond a binary prediction. Prospectively, these outputs could also be evaluated as aids to identifying cases in which ultrasound-based classification warrants particularly cautious interpretation or additional diagnostic assessment. The framework is interpretative rather than prescriptive; it does not constitute a patient-management algorithm and requires prospective evaluation before educational or clinical implementation.

### 4.8. Limitations

Several limitations should be considered. The study was exploratory and hypothesis-generating, and direct process-tracing measures such as eye tracking, verbal protocols, confidence ratings, or prospective decision experiments were not obtained. The model-derived structures therefore cannot determine how examiners prioritized or integrated features or whether they applied specific heuristic strategies. Similarly, signal detection measures quantify diagnostic discrimination and the observed decision criterion but do not establish the psychological reasons underlying the operating point. The panel comprised only six examiners, and experience, specialty, and training environment were closely intertwined; systematic comparisons across experience levels or professional subgroups were therefore not possible.

Model stability was assessed using stratified 5-fold cross-validation rather than extensive repeated cross-validation or bootstrap-based stability procedures. Internal resampling and bootstrap confidence intervals characterize within-cohort stability and uncertainty but do not establish external reproducibility across patient cohorts, examiner panels, institutions, ultrasound platforms, acquisition protocols, or time. Detailed fold-specific stability results and bootstrap confidence intervals are provided in Supplementary Tables S7A,B and S8 and Supplementary Figure S5. Although fold-specific root splits involved only boundary and tumor history, second-level ordering and feature retention varied. The typology should therefore be interpreted as a description of dominant upper-level patterns rather than as a set of fixed examiner-specific decision rules. Tree structures also depend on the settings and pruning behavior of the Decision Tree Learner, and deeper unpruned structures are particularly susceptible to sample-specific refinements.

Sonographic descriptors were aggregated to the case level by majority rating for the feature–reference-class association analyses. Although individual ratings were retained for the entropy-based disagreement analyses, majority aggregation did not preserve minority ratings in the corresponding association estimates. The weighted feature-use/reference-association alignment index was researcher-defined and represents one of several possible operationalizations rather than a validated or uniquely defined measure. Its relative stability in pruned trees, contrasted with its substantial sensitivity in unpruned trees, supports interpreting the index as an exploratory descriptive summary rather than as a measure of diagnostic performance, examiner cognition, decision quality, expertise, or the superiority of any cognitive strategy.

The examiners evaluated predefined ultrasound image sets rather than performing real-time examinations. This standardization ensured that all examiners assessed the same visual information, but it separated interpretation from acquisition and precluded adjustments to probe position or imaging settings, acquisition of additional planes, and dynamic reassessment of uncertain findings. The observed ratings, performance, and disagreement patterns may therefore not fully reflect routine real-time ultrasound practice; disagreement in descriptors such as boundary may partly depend on the information captured in the selected images. The direction of this effect remains uncertain because standardization may reduce acquisition-related variability, whereas the inability to obtain additional views may constrain performance. Prospective studies incorporating real-time ultrasound are required.

Finally, the study involved a modest retrospective single-center cohort of 149 histopathologically confirmed parotid lesions. A uniform histopathological reference standard is a methodological strength, but requiring tissue confirmation restricted the cohort to lesions that underwent invasive diagnostic verification or surgery and excluded conservatively managed lesions. This verification-related selection may have introduced spectrum bias by affecting disease prevalence, case complexity, and feature distributions. Consequently, the diagnostic performance, feature–reference-class association estimates, and model-derived structures may not generalize to unselected parotid-ultrasound populations. Independent prospective multicenter validation involving broader examiner populations and real-time ultrasound assessment is required.

## 5. Conclusions

This exploratory multimethod secondary analysis suggests that examiner-dependent benign/malignant classification in parotid ultrasound is not fully captured by diagnostic accuracy or interobserver agreement alone. Model-derived classification patterns were characterized by a small set of upper-level classification cues, particularly boundary and tumor history, which also showed the strongest cohort-level associations with the histopathological reference class. Disagreement in boundary assessment was also most strongly associated with divergent final classifications, identifying a potential target for prospective studies of descriptor standardization, descriptor-focused training and examiner feedback; whether improving consistency in boundary assessment would improve diagnostic accuracy remains to be tested. Signal detection analysis further distinguished examiner differences in diagnostic discrimination from differences in estimated within-cohort operating points, providing testable targets for prospective studies of examiner calibration, feedback, and training. Misleading-consensus cases illustrated that complete examiner agreement should not automatically be equated with diagnostic correctness when a coherent configuration of generally informative cues resembles the opposite histopathological reference class. Together, these findings define testable directions for prospective evaluation of descriptor-focused training, examiner feedback, and AI-based decision support intended to complement, rather than reproduce, existing human classification patterns.

## Figures and Tables

**Table 1:** Examiner-level performance and pruned surrogate-tree classification pattern.

Examiner	n Ratings	Pruned Classification Pattern	Pruned Tree Structure	Accuracy	Sensitivity	Specificity
Examiner 1	149	Context-based	Tumor History	81.2%	72.3%	85.3%
Examiner 2	149	Context-based	Tumor History	81.2%	68.1%	87.3%
Examiner 3	148	Morphology-based	Boundary	90.5%	85.1%	93.1%
Examiner 4	149	Hierarchical morphology-context	Boundary → Tumor History	77.9%	74.5%	79.4%
Examiner 5	149	Hierarchical morphology-context	Boundary → Tumor History	67.1%	72.3%	64.7%
Examiner 6	148	Hierarchical morphology-context	Boundary → Tumor History	63.5%	72.3%	59.4%

## Data Availability

The data are not publicly available due to privacy and ethical restrictions. De-identified data may be made available from the corresponding author upon reasonable request and subject to approval by the local ethics committee and applicable data protection regulations. Custom analysis scripts used for PMML parsing, post-processing, statistical analyses, table generation, and figure generation may be made available from the corresponding author upon reasonable request.

## Supplementary Materials

The following supporting information can be downloaded at: https://www.mdpi.com/article/10.3390/diagnostics16172793/s1, Figure S1: Tree-derived feature-use patterns in unpruned surrogate trees; Figure S2: Descriptor-specific associations with decision disagreement; Figure S3: Distribution of case-level decision-pattern groups; Figure S4: KNIME workflow for examiner-specific surrogate-tree generation and PMML export; Figure S5: Cross-validation-based root-split stability; Figure S6: Feature–reference-class association sensitivity across descriptor aggregation rules; Figure S7: Representative ultrasound images of the four misleading-consensus cases; Table S1: Cohort, age and tumor-history summaries, examiner characteristics, histopathology, and rating-completeness overview; Table S2: Surrogate-tree characteristics; Table S3: Tree-derived feature-use scores; Table S4: Case-level ambiguity metrics; Table S5: Descriptor-specific disagreement correlations; Table S6: Exploratory weighted feature-use/reference-association alignment index; Table S7A: Cross-validation root-split stability summary; Table S7B: Fold-specific pruned tree structures; Table S8: Bootstrap confidence intervals for examiner-level metrics; Table S9: Sensitivity of feature–reference-class associations across aggregation rules; Table S10: Feature–reference-class associations of clinical and sonographic features; Table S11: Weighting sensitivity of the exploratory weighted feature-use/reference-association alignment index; Table S12: Examiner-specific sonographic feature–reference associations; Table S13: Central effect estimates with case-bootstrap 95% confidence intervals.
