# Supplementary material for: Observable Classification Patterns and Diagnostic Uncertainty in Parotid Ultrasound: A Multimethod Secondary Analysis
Source: Diagnostics (Basel). 2026 Aug 30;16(17):2793. doi: 10.3390/diagnostics16172793 (PMC13565501; doi:10.3390/diagnostics16172793)
Supplement: Supplementary file 1 [file diagnostics-16-02793-s001.zip › Supplementary_Tables_S1-S13.pdf]

Supplementary Tables

Supplementary material index

Table	Content
Supplementary Table S1	Cohort, age and tumor-history summaries, examiner characteristics, histopathology, and rating-completeness overview
Supplementary Table S2	Surrogate-tree characteristics
Supplementary Table S3	Tree-derived feature-use scores
Supplementary Table S4	Case-level ambiguity metrics
Supplementary Table S5	Descriptor-specific disagreement correlations
Supplementary Table S6	Exploratory weighted feature-use/reference-association alignment index
Supplementary Tables S7A and S7B	Cross-validation root-split stability summary and fold-specific pruned tree structures
Supplementary Table S8	Bootstrap confidence intervals for examiner-level metrics
Supplementary Table S9	Sensitivity of feature–reference-class associations across aggregation rules
Supplementary Table S10	Feature–reference-class associations of clinical and sonographic features
Supplementary Table S11	Weighting sensitivity of the exploratory weighted feature-use/reference-association alignment index
Supplementary Table S12	Examiner-specific sonographic feature–reference associations
Supplementary Table S13	Central effect estimates with case-bootstrap 95% confidence intervals

Supplementary Table S1A. Cohort summary

Cohort-level summary derived from one row per case. For the age analysis, the histopathological reference class was coded as benign = 0 and malignant = 1. The percentile 95% confidence interval was obtained from 5000 case-level bootstrap resamples using NumPy's Generator with the PCG64 bit generator and seed 42.

Parameter	Value
Cases included	149
Expected examiner-case ratings	894
Available examiner-case ratings	892
Missing examiner-case ratings	2
Benign reference class, n (%)	102 (68.5%)
Malignant reference class, n (%)	47 (31.5%)
Age, median (IQR), years	64.0 (54.0–74.0)
Age among benign lesions, median (IQR), years	61.0 (53.0–67.0)
Age among malignant lesions, median (IQR), years	75.0 (59.0–83.0)
Age–reference-class association, point-biserial correlation (95% bootstrap CI)	0.314 (0.155–0.463)
Female, n (%)	68 (45.6%)
Male, n (%)	81 (54.4%)
Tumor history positive, n (%)	35 (23.5%)
Tumor history positive among benign lesions	10/102 (9.8%)
Tumor history positive among malignant lesions	25/47 (53.2%)

Supplementary Table S1B. Histopathological distribution

Histopathological findings and reference-class assignment.

Histopathological finding	Reference class	n	% of cases
malignant	Malignant	47	31.5
Warthin Tumor	Benign	41	27.5
Pleomorphic Adenoma	Benign	34	22.8
other	Benign	8	5.4
Sialadenitis	Benign	8	5.4
Lymph node	Benign	7	4.7
Cyst	Benign	3	2.0
Angioma	Benign	1	0.7

Supplementary Table S1C. Rating completeness

Expected and available examiner-case ratings.

Available ratings per case	Number of cases	% of cases
5	2	1.3
6	147	98.7
Expected ratings	894	
Available ratings	892	
Missing ratings	2	
Examiner 3 available ratings	148	One unavailable classification
Examiner 6 available ratings	148	One unavailable classification
Reason for isolated missing assessments	Not documented	
Analysis handling	Available-case analysis	No imputation

Supplementary Table S1D. Examiner characteristics

Confirmed clinical background and experience are reported descriptively; no specialty- or experience-subgroup inference was performed.

Examiner	Clinical background	Experience
Examiner 1	ENT specialist; DEGUM Level III	>20 years head-and-neck ultrasound
Examiner 2	ENT specialist; DEGUM Level II	19 years head-and-neck ultrasound
Examiner 3	ENT specialist	6 years head-and-neck ultrasound
Examiner 4	Radiation oncologist	>20 years head-and-neck imaging
Examiner 5	Final-year medical student	1 year head-and-neck imaging
Examiner 6	Neuroradiologist	7 years head-and-neck imaging

Supplementary Table S2. Surrogate-tree characteristics

Structural characteristics of pruned and unpruned examiner-specific surrogate trees.

Examiner	Tree type	n cases in tree	Internal split nodes	Leaves	Total nodes	Maximum depth	Root split	Top 3 weighted features
Examiner 1	Pruned	149	1	2	3	1	Tumor History	Tumor History (100.0%)
Examiner 1	Unpruned	149	28	38	66	10	Tumor History	Tumor History (48.1%); Boundary (21.2%); Vascularization (9.6%)
Examiner 2	Pruned	149	1	2	3	1	Tumor History	Tumor History (100.0%)
Examiner 2	Unpruned	149	28	39	67	9	Tumor History	Tumor History (52.8%); Boundary (20.3%); Vascularization (7.0%)
Examiner 3	Pruned	148	1	2	3	1	Boundary	Boundary (100.0%)
Examiner 3	Unpruned	148	33	42	75	8	Boundary	Boundary (44.7%); Tumor History (16.0%); Acoustic Features (9.6%)
Examiner 4	Pruned	149	2	3	5	2	Boundary	Boundary (76.0%); Tumor History (24.0%)
Examiner 4	Unpruned	149	27	37	64	10	Boundary	Boundary (48.0%); Tumor History (16.9%); Nicotine Consumption (11.8%)
Examiner 5	Pruned	149	2	3	5	2	Boundary	Boundary (77.0%); Tumor History (23.0%)
Examiner 5	Unpruned	149	28	36	64	10	Boundary	Boundary (44.2%); Facial Nerve Palsy (16.3%); Tumor History (13.4%)
Examiner 6	Pruned	148	2	3	5	2	Boundary	Boundary (74.6%); Tumor History (25.4%)
Examiner 6	Unpruned	148	45	60	105	10	Boundary	Boundary (42.2%); Facial Nerve Palsy (14.4%); Number of Tumors (9.7%)

Supplementary Table S3. Tree-derived feature-use scores

Feature-use scores derived from pruned and unpruned surrogate-tree split nodes. Feature–reference-class association statistics and retained upper-level cue status are reported in Supplementary Table S10.

Examiner	Tree type	Feature	Feature type	Split count	Earliest depth	Weighted score	Relative share
Examiner 1	Pruned	Tumor History	Clinical context	1	0	1	1
Examiner 2	Pruned	Tumor History	Clinical context	1	0	1	1
Examiner 3	Pruned	Boundary	Sonographic morphology	1	0	1	1
Examiner 4	Pruned	Boundary	Sonographic morphology	1	0	1	0.76
Examiner 4	Pruned	Tumor History	Clinical context	1	1	0.315	0.24
Examiner 5	Pruned	Boundary	Sonographic morphology	1	0	1	0.77
Examiner 5	Pruned	Tumor History	Clinical context	1	1	0.299	0.23
Examiner 6	Pruned	Boundary	Sonographic morphology	1	0	1	0.746
Examiner 6	Pruned	Tumor History	Clinical context	1	1	0.341	0.254
Examiner 1	Unpruned	Tumor History	Clinical context	1	0	1	0.481
Examiner 1	Unpruned	Boundary	Sonographic morphology	2	1	0.441	0.212
Examiner 1	Unpruned	Vascularization	Sonographic morphology	2	1	0.2	0.096
Examiner 1	Unpruned	Echogenicity	Sonographic morphology	2	2	0.199	0.096
Examiner 1	Unpruned	Acoustic Features	Sonographic morphology	3	3	0.06	0.029
Examiner 1	Unpruned	Number of Tumors	Sonographic morphology	5	3	0.06	0.029
Examiner 1	Unpruned	Texture	Sonographic morphology	4	3	0.044	0.021
Examiner 1	Unpruned	Nicotine Consumption	Clinical context	5	3	0.043	0.021
Examiner 1	Unpruned	Contour	Sonographic morphology	2	4	0.026	0.013
Examiner 1	Unpruned	Facial Nerve Palsy	Clinical context	2	9	0.005	0.002
Examiner 2	Unpruned	Tumor History	Clinical context	1	0	1	0.528
Examiner 2	Unpruned	Boundary	Sonographic morphology	2	1	0.384	0.203
Examiner 2	Unpruned	Vascularization	Sonographic morphology	2	1	0.132	0.07
Examiner 2	Unpruned	Nicotine Consumption	Clinical context	3	2	0.099	0.052
Examiner 2	Unpruned	Facial Nerve Palsy	Clinical context	1	2	0.078	0.041
Examiner 2	Unpruned	Texture	Sonographic morphology	3	3	0.057	0.03
Examiner 2	Unpruned	Number of Tumors	Sonographic morphology	6	3	0.042	0.022
Examiner 2	Unpruned	Echogenicity	Sonographic morphology	2	3	0.036	0.019
Examiner 2	Unpruned	Contour	Sonographic morphology	5	4	0.035	0.018
Examiner 2	Unpruned	Acoustic Features	Sonographic morphology	3	4	0.031	0.016
Examiner 3	Unpruned	Boundary	Sonographic morphology	1	0	1	0.447
Examiner 3	Unpruned	Tumor History	Clinical context	3	1	0.357	0.16
Examiner 3	Unpruned	Acoustic Features	Sonographic morphology	6	2	0.214	0.096
Examiner 3	Unpruned	Contour	Sonographic morphology	2	1	0.188	0.084
Examiner 3	Unpruned	Texture	Sonographic morphology	2	3	0.129	0.058
Examiner 3	Unpruned	Echogenicity	Sonographic morphology	4	2	0.123	0.055
Examiner 3	Unpruned	Vascularization	Sonographic morphology	8	2	0.101	0.045
Examiner 3	Unpruned	Nicotine Consumption	Clinical context	3	4	0.053	0.024
Examiner 3	Unpruned	Number of Tumors	Sonographic morphology	2	2	0.039	0.017
Examiner 3	Unpruned	Facial Nerve Palsy	Clinical context	2	3	0.033	0.015
Examiner 4	Unpruned	Boundary	Sonographic morphology	1	0	1	0.48
Examiner 4	Unpruned	Tumor History	Clinical context	3	1	0.353	0.169
Examiner 4	Unpruned	Nicotine Consumption	Clinical context	3	2	0.246	0.118
Examiner 4	Unpruned	Facial Nerve Palsy	Clinical context	1	1	0.185	0.089
Examiner 4	Unpruned	Texture	Sonographic morphology	2	2	0.109	0.052
Examiner 4	Unpruned	Echogenicity	Sonographic morphology	4	3	0.074	0.036
Examiner 4	Unpruned	Vascularization	Sonographic morphology	4	2	0.062	0.03
Examiner 4	Unpruned	Acoustic Features	Sonographic morphology	3	4	0.031	0.015
Examiner 4	Unpruned	Number of Tumors	Sonographic morphology	4	4	0.016	0.008
Examiner 4	Unpruned	Contour	Sonographic morphology	2	6	0.01	0.005
Examiner 5	Unpruned	Boundary	Sonographic morphology	1	0	1	0.442
Examiner 5	Unpruned	Facial Nerve Palsy	Clinical context	2	1	0.369	0.163
Examiner 5	Unpruned	Tumor History	Clinical context	2	1	0.304	0.134
Examiner 5	Unpruned	Contour	Sonographic morphology	2	3	0.198	0.087
Examiner 5	Unpruned	Number of Tumors	Sonographic morphology	3	2	0.129	0.057
Examiner 5	Unpruned	Acoustic Features	Sonographic morphology	4	4	0.097	0.043
Examiner 5	Unpruned	Nicotine Consumption	Clinical context	5	2	0.082	0.036
Examiner 5	Unpruned	Texture	Sonographic morphology	4	5	0.036	0.016
Examiner 5	Unpruned	Echogenicity	Sonographic morphology	2	3	0.028	0.012
Examiner 5	Unpruned	Vascularization	Sonographic morphology	3	5	0.021	0.009
Examiner 6	Unpruned	Boundary	Sonographic morphology	1	0	1	0.422
Examiner 6	Unpruned	Facial Nerve Palsy	Clinical context	1	1	0.341	0.144
Examiner 6	Unpruned	Number of Tumors	Sonographic morphology	2	2	0.23	0.097
Examiner 6	Unpruned	Nicotine Consumption	Clinical context	3	2	0.224	0.095
Examiner 6	Unpruned	Acoustic Features	Sonographic morphology	9	1	0.179	0.076
Examiner 6	Unpruned	Tumor History	Clinical context	4	3	0.141	0.059
Examiner 6	Unpruned	Echogenicity	Sonographic morphology	8	3	0.119	0.05
Examiner 6	Unpruned	Vascularization	Sonographic morphology	7	2	0.056	0.024
Examiner 6	Unpruned	Texture	Sonographic morphology	4	3	0.049	0.021
Examiner 6	Unpruned	Contour	Sonographic morphology	6	4	0.029	0.012

Supplementary Table S4. Case-level ambiguity metrics

Compact case-level table showing decision disagreement, overall descriptor disagreement, error burden, and case type. For each descriptor and case, $H = -\Sigma p_i \log(p_i)$ was normalized as E_occ = H/log(K_observed), where K_observed was the number of categories with at least one available rating; E_occ = 0 when K_observed ≤ 1. Overall descriptor disagreement is the mean E_occ across eight descriptors.

Case	Reference class	n ratings	Malignant ratings	p malignant	Decision disagreement	Error burden	Descriptor disagreement	Case type	Boundary disagreement
1	malignant	6	5	0.833	0.65	0.167	0.473	Partial disagreement	0.65
2	benign	6	0	0	0	0	0.196	Correct consensus	0
3	benign	6	0	0	0	0	0.196	Correct consensus	0
4	benign	6	3	0.5	1	0.5	0.572	Maximal disagreement	0.918
5	benign	6	0	0	0	0	0.426	Correct consensus	0
6	malignant	6	5	0.833	0.65	0.167	0.713	Partial disagreement	0.65
7	benign	6	4	0.667	0.918	0.667	0.474	Partial disagreement	0.65
8	benign	6	5	0.833	0.65	0.833	0.355	Partial disagreement	0.918
9	malignant	6	5	0.833	0.65	0.167	0.655	Partial disagreement	0.918
10	benign	6	2	0.333	0.918	0.333	0.402	Partial disagreement	0.918
11	benign	6	2	0.333	0.918	0.333	0.527	Partial disagreement	0.65
12	malignant	6	2	0.333	0.918	0.667	0.454	Partial disagreement	0
13	benign	6	1	0.167	0.65	0.167	0.616	Partial disagreement	1
14	benign	6	0	0	0	0	0.277	Correct consensus	0.65
15	malignant	6	5	0.833	0.65	0.167	0.728	Partial disagreement	0.65
16	malignant	6	4	0.667	0.918	0.333	0.359	Partial disagreement	0
17	malignant	6	6	1	0	0	0.632	Correct consensus	0
18	malignant	6	6	1	0	0	0.501	Correct consensus	1
19	benign	6	1	0.167	0.65	0.167	0.081	Partial disagreement	0
20	malignant	6	5	0.833	0.65	0.167	0.288	Partial disagreement	0.65
21	benign	6	0	0	0	0	0.392	Correct consensus	0
22	benign	6	1	0.167	0.65	0.167	0.311	Partial disagreement	0.65
23	benign	6	1	0.167	0.65	0.167	0.555	Partial disagreement	0.65
24	benign	6	2	0.333	0.918	0.333	0.66	Partial disagreement	0.918
25	benign	6	0	0	0	0	0.555	Correct consensus	0
26	benign	6	0	0	0	0	0.295	Correct consensus	0
27	malignant	6	6	1	0	0	0.163	Correct consensus	0
28	benign	6	5	0.833	0.65	0.833	0.484	Partial disagreement	0.65
29	malignant	6	4	0.667	0.918	0.333	0.196	Partial disagreement	0
30	malignant	6	1	0.167	0.65	0.833	0.311	Partial disagreement	0
31	benign	6	1	0.167	0.65	0.167	0.261	Partial disagreement	0
32	benign	6	3	0.5	1	0.5	0.355	Maximal disagreement	0
33	malignant	6	5	0.833	0.65	0.167	0.703	Partial disagreement	0.65
34	benign	6	0	0	0	0	0.565	Correct consensus	0
35	benign	6	2	0.333	0.918	0.333	0.413	Partial disagreement	0.65
36	benign	5	2	0.4	0.971	0.4	0.428	Partial disagreement	0
37	malignant	6	4	0.667	0.918	0.333	0.473	Partial disagreement	0
38	benign	6	2	0.333	0.918	0.333	0.561	Partial disagreement	0.918
39	malignant	6	1	0.167	0.65	0.833	0.703	Partial disagreement	1
40	benign	6	2	0.333	0.918	0.333	0.848	Partial disagreement	0.918
41	benign	6	1	0.167	0.65	0.167	0.392	Partial disagreement	0
42	malignant	6	5	0.833	0.65	0.167	0.23	Partial disagreement	0
43	benign	6	1	0.167	0.65	0.167	0.277	Partial disagreement	0.65
44	benign	6	0	0	0	0	0.125	Correct consensus	0
45	benign	6	1	0.167	0.65	0.167	0.517	Partial disagreement	0
46	benign	6	1	0.167	0.65	0.167	0.561	Partial disagreement	0
47	benign	6	1	0.167	0.65	0.167	0.68	Partial disagreement	0.65
48	benign	6	0	0	0	0	0.474	Correct consensus	0.65
49	malignant	6	0	0	0	1	0.325	Misleading consensus	0
50	benign	6	0	0	0	0	0.325	Correct consensus	0
51	benign	6	4	0.667	0.918	0.667	0.703	Partial disagreement	0.65
52	benign	6	1	0.167	0.65	0.167	0.616	Partial disagreement	0.918
53	benign	6	0	0	0	0	0.376	Correct consensus	0
54	benign	6	3	0.5	1	0.5	0.616	Maximal disagreement	0.918
55	benign	6	5	0.833	0.65	0.833	0.539	Partial disagreement	0.65
56	benign	6	0	0	0	0	0.632	Correct consensus	0.65
57	malignant	6	6	1	0	0	0.359	Correct consensus	0
58	benign	6	2	0.333	0.918	0.333	0.342	Partial disagreement	0.65
59	benign	5	0	0	0	0	0.576	Correct consensus	0
60	benign	6	0	0	0	0	0.616	Correct consensus	0.918
61	malignant	6	1	0.167	0.65	0.833	0.42	Partial disagreement	0
62	benign	6	1	0.167	0.65	0.167	0.517	Partial disagreement	0
63	malignant	6	1	0.167	0.65	0.833	0.501	Partial disagreement	0
64	malignant	6	4	0.667	0.918	0.333	0.345	Partial disagreement	0
65	malignant	6	6	1	0	0	0.555	Correct consensus	0
66	malignant	6	2	0.333	0.918	0.667	0.592	Partial disagreement	1
67	malignant	6	4	0.667	0.918	0.333	0.474	Partial disagreement	0.918
68	malignant	6	4	0.667	0.918	0.333	0.355	Partial disagreement	0
69	benign	6	6	1	0	1	0.471	Misleading consensus	0
70	benign	6	2	0.333	0.918	0.333	0.413	Partial disagreement	0
71	malignant	6	6	1	0	0	0.484	Correct consensus	0
72	malignant	6	6	1	0	0	0.501	Correct consensus	0.65
73	malignant	6	5	0.833	0.65	0.167	0.527	Partial disagreement	0.65
74	malignant	6	6	1	0	0	0.424	Correct consensus	0

Case	Reference class	n ratings	Malignant ratings	p malignant	Decision disagreement	Error burden	Descriptor disagreement	Case type	Boundary disagreement
75	malignant	6	4	0.667	0.918	0.333	0.67	Partial disagreement	0.65
76	benign	6	0	0	0	0	0.376	Correct consensus	0
77	benign	6	1	0.167	0.65	0.167	0.484	Partial disagreement	0
78	malignant	6	5	0.833	0.65	0.167	0.393	Partial disagreement	0
79	malignant	6	6	1	0	0	0.459	Correct consensus	0
80	benign	6	1	0.167	0.65	0.167	0.436	Partial disagreement	0
81	malignant	6	4	0.667	0.918	0.333	0.359	Partial disagreement	0
82	malignant	6	3	0.5	1	0.5	0.606	Maximal disagreement	0
83	benign	6	1	0.167	0.65	0.167	0.673	Partial disagreement	0
84	benign	6	0	0	0	0	0.473	Correct consensus	0
85	benign	6	0	0	0	0	0.392	Correct consensus	0
86	benign	6	0	0	0	0	0.359	Correct consensus	0
87	malignant	6	5	0.833	0.65	0.167	0.551	Partial disagreement	0.918
88	benign	6	0	0	0	0	0.305	Correct consensus	0
89	malignant	6	5	0.833	0.65	0.167	0.081	Partial disagreement	0
90	malignant	6	6	1	0	0	0.556	Correct consensus	0
91	benign	6	0	0	0	0	0.402	Correct consensus	0
92	benign	6	0	0	0	0	0.666	Correct consensus	0.65
93	malignant	6	5	0.833	0.65	0.167	0.426	Partial disagreement	0
94	benign	6	1	0.167	0.65	0.167	0.578	Partial disagreement	0
95	benign	6	2	0.333	0.918	0.333	0.501	Partial disagreement	0
96	benign	6	3	0.5	1	0.5	0.517	Maximal disagreement	0.918
97	benign	6	0	0	0	0	0.393	Correct consensus	0
98	benign	6	0	0	0	0	0.459	Correct consensus	0
99	benign	6	0	0	0	0	0.555	Correct consensus	0
100	benign	6	0	0	0	0	0.196	Correct consensus	0
101	benign	6	1	0.167	0.65	0.167	0.517	Partial disagreement	0
102	malignant	6	5	0.833	0.65	0.167	0.565	Partial disagreement	1
103	benign	6	0	0	0	0	0.321	Correct consensus	0
104	benign	6	1	0.167	0.65	0.167	0.44	Partial disagreement	0.65
105	benign	6	1	0.167	0.65	0.167	0.565	Partial disagreement	0
106	benign	6	1	0.167	0.65	0.167	0.288	Partial disagreement	0.65
107	benign	6	4	0.667	0.918	0.667	0.081	Partial disagreement	0
108	benign	6	0	0	0	0	0.561	Correct consensus	0
109	benign	6	0	0	0	0	0.426	Correct consensus	0
110	benign	6	0	0	0	0	0.632	Correct consensus	0.65
111	benign	6	5	0.833	0.65	0.833	0.713	Partial disagreement	0.65
112	malignant	6	6	1	0	0	0.501	Correct consensus	0.65
113	benign	6	0	0	0	0	0.507	Correct consensus	0
114	malignant	6	6	1	0	0	0.588	Correct consensus	0
115	benign	6	1	0.167	0.65	0.167	0.359	Partial disagreement	0.65
116	benign	6	5	0.833	0.65	0.833	0.392	Partial disagreement	0
117	benign	6	0	0	0	0	0.345	Correct consensus	0
118	benign	6	0	0	0	0	0.196	Correct consensus	0
119	malignant	6	6	1	0	0	0.436	Correct consensus	0.65
120	benign	6	6	1	0	1	0.277	Misleading consensus	0.65
121	malignant	6	1	0.167	0.65	0.833	0.402	Partial disagreement	0.65
122	benign	6	5	0.833	0.65	0.833	0.402	Partial disagreement	0.65
123	benign	6	2	0.333	0.918	0.333	0.703	Partial disagreement	0.65
124	benign	6	2	0.333	0.918	0.333	0.68	Partial disagreement	0.918
125	benign	6	1	0.167	0.65	0.167	0.163	Partial disagreement	0
126	benign	6	0	0	0	0	0.369	Correct consensus	0
127	benign	6	6	1	0	1	0.406	Misleading consensus	0.65
128	malignant	6	6	1	0	0	0.338	Correct consensus	0.918
129	benign	6	0	0	0	0	0.355	Correct consensus	0
130	benign	6	2	0.333	0.918	0.333	0.555	Partial disagreement	0.918
131	benign	6	0	0	0	0	0.206	Correct consensus	0
132	malignant	6	4	0.667	0.918	0.333	0.311	Partial disagreement	0.65
133	benign	6	1	0.167	0.65	0.167	0.277	Partial disagreement	0
134	benign	6	1	0.167	0.65	0.167	0.684	Partial disagreement	0.65
135	benign	6	1	0.167	0.65	0.167	0.517	Partial disagreement	0
136	malignant	6	6	1	0	0	0.244	Correct consensus	0.65
137	benign	6	0	0	0	0	0.081	Correct consensus	0
138	benign	6	1	0.167	0.65	0.167	0.521	Partial disagreement	0.65
139	benign	6	2	0.333	0.918	0.333	0.565	Partial disagreement	0.65
140	benign	6	1	0.167	0.65	0.167	0.376	Partial disagreement	0.65
141	malignant	6	6	1	0	0	0.369	Correct consensus	0
142	benign	6	1	0.167	0.65	0.167	0.724	Partial disagreement	0
143	benign	6	3	0.5	1	0.5	0.616	Maximal disagreement	0.918
144	benign	6	0	0	0	0	0.244	Correct consensus	0
145	benign	6	0	0	0	0	0.484	Correct consensus	0
146	benign	6	1	0.167	0.65	0.167	0.588	Partial disagreement	0.65
147	benign	6	0	0	0	0	0.321	Correct consensus	0
148	benign	6	1	0.167	0.65	0.167	0.321	Partial disagreement	0
149	benign	6	0	0	0	0	0.163	Correct consensus	0

Supplementary Table S5. Descriptor disagreement correlations

Descriptor-specific Spearman correlations with decision disagreement and error burden, together with mean E_occ. E_occ = H/log(K_observed), with K_observed defined per descriptor and case as the number of occupied rating categories and E_occ = 0 when K_observed ≤ 1. Algebraically identical entropy values were rounded to 12 decimal places before rank transformation.

Descriptor	Spearman ρ with decision disagreement	p value with decision disagreement	Spearman ρ with error burden	p value with error burden	Mean descriptor entropy
Boundary	0.361	6.20e-06	0.348	1.38e-05	0.322
Number of Tumors	0.206	0.012	0.102	0.216	0.257
Texture	0.136	0.098	0.093	0.258	0.462
Acoustic Features	0.105	0.202	0.111	0.179	0.497
Contour	0.100	0.226	0.040	0.627	0.599
Echogenicity	0.013	0.871	-0.023	0.780	0.705
Size	0.003	0.974	0.010	0.903	0.240
Vascularization	-0.023	0.781	-0.042	0.608	0.492

Supplementary Table S6. Exploratory weighted feature-use/reference-association alignment index

Weighted feature-use/reference-association alignment index and clinical-context versus sonographic-morphology shares by examiner and tree type. Weighted feature-use/reference-association alignment indices were calculated using unrounded Cramér's V values and rounded to three decimals for presentation.

Examiner	Tree type	Weighted alignment index	n_features_used	context_share	morphology_share
Examiner 1	Pruned	0.476	1	1	0
Examiner 1	Unpruned	0.387	10	0.504	0.496
Examiner 2	Pruned	0.476	1	1	0
Examiner 2	Unpruned	0.390	10	0.622	0.378
Examiner 3	Pruned	0.435	1	0	1
Examiner 3	Unpruned	0.342	10	0.198	0.802
Examiner 4	Pruned	0.445	2	0.24	0.76
Examiner 4	Unpruned	0.370	10	0.376	0.624
Examiner 5	Pruned	0.444	2	0.23	0.77
Examiner 5	Unpruned	0.332	10	0.334	0.666
Examiner 6	Pruned	0.445	2	0.254	0.746
Examiner 6	Unpruned	0.308	10	0.298	0.702

Supplementary Table S7A. Cross-validation root-split stability summary

Summary of root-split stability across stratified 5-fold cross-validation of pruned surrogate trees.

Examiner	Full-data pruned structure	Full-data root split	Most frequent CV root split	Most frequent CV root split frequency	Full-data root split reproduced	Tumor History as root split	Boundary as root split	Tumor History used anywhere	Boundary used anywhere	Boundary and Tumor History both used	Most frequent fold-specific upper-level tree pattern	Pattern frequency
Examiner 1	Tumor History	Tumor History	Tumor History	3/5	3/5	3/5	2/5	4/5	2/5	1/5	Tumor-history-rooted	3/5
Examiner 2	Tumor History	Tumor History	Tumor History	4/5	4/5	4/5	1/5	4/5	1/5	0/5	Tumor-history-rooted	4/5
Examiner 3	Boundary	Boundary	Boundary	5/5	5/5	0/5	5/5	0/5	5/5	0/5	Boundary-rooted	5/5
Examiner 4	Boundary → Tumor History	Boundary	Boundary	4/5	4/5	1/5	4/5	4/5	5/5	4/5	Boundary-rooted with tumor-history refinement	3/5
Examiner 5	Boundary → Tumor History	Boundary	Boundary	5/5	5/5	0/5	5/5	2/5	5/5	2/5	Boundary-rooted	3/5
Examiner 6	Boundary → Tumor History	Boundary	Boundary	5/5	5/5	0/5	5/5	4/5	5/5	4/5	Boundary-rooted with tumor-history refinement	4/5

Supplementary Table S7B. Fold-specific pruned tree structures

Detailed fold-specific tree structures from the cross-validation-based stability analysis.

Examiner	Fold	Training cases	Root split	Second-level split features	All split features	Internal split nodes	Leaves	Total nodes	Maximum depth	Fold-specific upper-level tree pattern
Examiner 1	1	119	Boundary	Tumor History	Boundary; Tumor History	2	3	5	2	Boundary-rooted with tumor-history refinement
Examiner 1	2	119	Boundary	—	Boundary	1	2	3	1	Boundary-rooted
Examiner 1	3	119	Tumor History	—	Tumor History	1	2	3	1	Tumor-history-rooted
Examiner 1	4	119	Tumor History	—	Tumor History	1	2	3	1	Tumor-history-rooted
Examiner 1	5	120	Tumor History	—	Tumor History	1	2	3	1	Tumor-history-rooted
Examiner 2	1	119	Boundary	—	Boundary	1	2	3	1	Boundary-rooted
Examiner 2	2	119	Tumor History	—	Tumor History	1	2	3	1	Tumor-history-rooted
Examiner 2	3	119	Tumor History	—	Tumor History	1	2	3	1	Tumor-history-rooted
Examiner 2	4	119	Tumor History	—	Tumor History	1	2	3	1	Tumor-history-rooted
Examiner 2	5	120	Tumor History	—	Tumor History	1	2	3	1	Tumor-history-rooted
Examiner 3	1	118	Boundary	—	Boundary	1	2	3	1	Boundary-rooted
Examiner 3	2	118	Boundary	—	Boundary	1	2	3	1	Boundary-rooted
Examiner 3	3	118	Boundary	—	Boundary	1	2	3	1	Boundary-rooted
Examiner 3	4	119	Boundary	—	Boundary	1	2	3	1	Boundary-rooted
Examiner 3	5	119	Boundary	—	Boundary	1	2	3	1	Boundary-rooted
Examiner 4	1	119	Tumor History	Boundary	Tumor History; Boundary	2	3	5	2	Tumor-history-rooted with boundary refinement
Examiner 4	2	119	Boundary	Tumor History	Boundary; Tumor History	2	3	5	2	Boundary-rooted with tumor-history refinement
Examiner 4	3	119	Boundary	Tumor History	Boundary; Tumor History	2	3	5	2	Boundary-rooted with tumor-history refinement
Examiner 4	4	119	Boundary	Tumor History	Boundary; Tumor History	2	3	5	2	Boundary-rooted with tumor-history refinement
Examiner 4	5	120	Boundary	—	Boundary	1	2	3	1	Boundary-rooted
Examiner 5	1	119	Boundary	Tumor History	Boundary; Tumor History	2	3	5	2	Boundary-rooted with tumor-history refinement
Examiner 5	2	119	Boundary	—	Boundary	1	2	3	1	Boundary-rooted
Examiner 5	3	119	Boundary	—	Boundary	1	2	3	1	Boundary-rooted
Examiner 5	4	119	Boundary	Tumor History	Boundary; Tumor History	2	3	5	2	Boundary-rooted with tumor-history refinement
Examiner 5	5	120	Boundary	—	Boundary	1	2	3	1	Boundary-rooted
Examiner 6	1	118	Boundary	Tumor History	Boundary; Tumor History	2	3	5	2	Boundary-rooted with tumor-history refinement
Examiner 6	2	118	Boundary	—	Boundary	1	2	3	1	Boundary-rooted
Examiner 6	3	118	Boundary	Tumor History	Boundary; Tumor History	2	3	5	2	Boundary-rooted with tumor-history refinement
Examiner 6	4	119	Boundary	Tumor History	Boundary; Tumor History	2	3	5	2	Boundary-rooted with tumor-history refinement

Examiner	Fold	Training cases	Root split	Second-level split features	All split features	Internal split nodes	Leaves	Total nodes	Maximum depth	Fold-specific upper-level tree pattern
Examiner 6	5	119	Boundary	Tumor History	Boundary; Tumor History	2	3	5	2	Boundary-rooted with tumor-history refinement

Supplementary Table S8. Bootstrap confidence intervals for examiner-level metrics

Diagnostic performance and signal detection metrics with percentile 95% confidence intervals from case-level bootstrap resampling. Bootstrap samples were generated with NumPy’s Generator using the PCG64 bit generator and seed 42. Confidence limits were the 2.5th and 97.5th percentiles calculated using linear interpolation.

Examiner	n ratings	TP	FP	FN	TN	Accuracy	Sensitivity	Specificity	PPV	NPV	d′	c
Examiner 1	149	34	15	13	87	81.2% [74.5–87.2%]	72.3% [59.2–84.6%]	85.3% [78.2–91.8%]	69.4% [56.2–82.2%]	87.0% [79.8–93.1%]	1.64 [1.18–2.19]	0.23 [-0.02–0.48]
Examiner 2	149	32	13	15	89	81.2% [74.5–87.2%]	68.1% [54.2–81.1%]	87.3% [80.6–93.4%]	71.1% [57.4–84.0%]	85.6% [78.7–91.9%]	1.61 [1.15–2.16]	0.33 [0.09–0.59]
Examiner 3	148	40	7	7	94	90.5% [85.8–94.6%]	85.1% [74.4–94.4%]	93.1% [87.8–97.3%]	85.1% [74.4–94.3%]	93.1% [87.8–97.8%]	2.52 [2.01–3.27]	0.22 [-0.09–0.53]
Examiner 4	149	35	21	12	81	77.9% [71.1–84.6%]	74.5% [61.5–86.4%]	79.4% [71.4–87.0%]	62.5% [50.0–75.0%]	87.1% [80.0–93.6%]	1.48 [1.03–2.02]	0.08 [-0.17–0.31]
Examiner 5	149	34	36	13	66	67.1% [59.7–74.5%]	72.3% [59.5–84.6%]	64.7% [55.1–73.7%]	48.6% [36.5–60.3%]	83.5% [75.0–91.1%]	0.97 [0.54–1.47]	-0.11 [-0.35–0.11]
Examiner 6	148	34	41	13	60	63.5% [55.4–71.6%]	72.3% [59.5–84.8%]	59.4% [49.5–69.1%]	45.3% [33.8–56.8%]	82.2% [73.0–90.5%]	0.83 [0.39–1.34]	-0.18 [-0.42–0.04]

Supplementary Table S9. Sensitivity of feature–reference-class associations across aggregation rules

Sensitivity of feature–reference associations under A0 (>50% of available ratings), A1 (at least four concordant ratings), and A2 (at least five concordant ratings). Majority aggregation is a case-level cohort summary that can obscure examiner-specific variation; Supplementary Table S12 provides the corresponding examiner-specific sonographic sensitivity analysis. Benjamini–Hochberg FDR adjustment was performed separately across the 14 feature–reference-class association tests within each aggregation rule.

Aggregation approach	Aggregation rule	Top overall feature	Top overall Cramér's V	Top sonographic feature	Top sonographic Cramér's V	Tumor History Cramér's V	Tumor History FDR p	Boundary Cramér's V	Boundary FDR p	Boundary evaluable cases	Echogenicity Cramér's V	Echogenicity FDR p	Main conclusion
Main majority aggregation	Top category >50% of available examiner ratings	Tumor History	0.476	Boundary	0.435	0.476	9.00e-08	0.435	1.26e-06	144	0.385	0.003	Boundary remained the top sonographic feature
Strict aggregation ≥4 ratings	Top category supported by at least 4 examiner ratings	Tumor History	0.476	Boundary	0.435	0.476	9.00e-08	0.435	1.26e-06	144	0.382	0.003	Boundary remained the top sonographic feature
High-consensus aggregation ≥5 ratings	Top category supported by at least 5 examiner ratings	Boundary	0.484	Boundary	0.484	0.476	9.00e-08	0.484	3.47e-07	127	0.292	0.116	Boundary remained the top sonographic feature

Supplementary Table S10. Feature–reference-class associations of clinical and sonographic features

Complete feature–reference-class association results from the main majority-aggregation analysis, including evaluable case counts, Cramér's V, unadjusted p values, FDR-adjusted p values, and interpretation.

Feature	Feature type	Evaluable cases	Cramér's V	p value	FDR-adjusted p value	Interpretation
Tumor History	Clinical context	149	0.476	<0.001	<0.001	Retained upper-level cue
Boundary	Sonographic morphology	144	0.435	<0.001	<0.001	Retained upper-level cue
Echogenicity	Sonographic morphology	118	0.385	<0.001	0.003	Significant
Nicotine Consumption	Clinical context	149	0.259	0.007	0.023	Significant
Facial Nerve Palsy	Clinical context	149	0.224	0.010	0.028	Significant
Texture	Sonographic morphology	136	0.183	0.033	0.076	Not significant after FDR correction
Alcohol Consumption	Clinical context	149	0.172	0.222	0.283	Not significant
Contour	Sonographic morphology	118	0.172	0.173	0.270	Not significant
Size	Sonographic morphology	145	0.162	0.149	0.260	Not significant
B Symptoms	Clinical context	149	0.155	0.093	0.186	Not significant
Vascularization	Sonographic morphology	128	0.142	0.277	0.298	Not significant
Gender	Clinical context	149	0.100	0.222	0.283	Not significant
Acoustic Features	Sonographic morphology	129	0.099	0.260	0.298	Not significant
Number of Tumors	Sonographic morphology	143	0.030	0.721	0.721	Not significant

Table note: Cramér's V quantifies association strength. Benjamini–Hochberg FDR adjustment was applied across the 14 categorical feature–reference-class association tests reported in this table, which were treated as a single test family. FDR = false discovery rate.

Supplementary Table S11. Weighting sensitivity of the exploratory weighted feature-use/reference-association alignment index

W0 = node support × 1/(depth+1); W1 = unweighted split frequency; W2 = node support without depth weighting; W3 = node support × 2^(−depth). Values are weighted alignment indices after within-tree normalization and are rounded for presentation. Rank comparisons are descriptive and were not tested for significance. 'Top feature stable = No' may reflect a tie under at least one weighting scheme and does not necessarily indicate replacement by a different uniquely top-ranked feature.

**Examiner**	**Tree**	**W0**	**W1**	**W2**	**W3**	**Max |Δ| vs W0 (%)**	**Max examiner-rank shift**	**Top feature stable**	**Top-2 stable**
Examiner 1	Pruned	0.476	0.476	0.476	0.476	0.00	0.0	Yes	Yes
Examiner 2	Pruned	0.476	0.476	0.476	0.476	0.00	0.0	Yes	Yes
Examiner 3	Pruned	0.435	0.435	0.435	0.435	0.00	0.0	Yes	Yes
Examiner 4	Pruned	0.445	0.455	0.451	0.445	2.38	0.0	No	Yes
Examiner 5	Pruned	0.444	0.455	0.450	0.444	2.47	1.0	No	Yes
Examiner 6	Pruned	0.445	0.455	0.451	0.445	2.24	1.0	No	Yes
Examiner 1	Unpruned	0.387	0.202	0.322	0.411	47.73	3.0	No	No
Examiner 2	Unpruned	0.390	0.189	0.312	0.416	51.60	5.0	No	No
Examiner 3	Unpruned	0.342	0.216	0.282	0.367	36.92	1.0	No	No

Examiner	Tree	W0	W1	W2	W3	Max |Δ| vs W0 (%)	Max examiner-rank shift	Top feature stable	Top-2 stable
Examiner 4	Unpruned	0.370	0.226	0.323	0.386	38.87	2.0	No	No
Examiner 5	Unpruned	0.332	0.210	0.264	0.362	36.61	1.0	No	No
Examiner 6	Unpruned	0.308	0.225	0.271	0.317	26.83	4.0	No	No

Supplementary Table S12. Examiner-specific sonographic feature–reference associations

Cells show Cramér's V for each examiner's original descriptor rating versus the binary histopathological reference. These are sensitivity analyses of feature–reference associations, not measurements of examiner cognition or feature attention. The A0 majority Boundary value was 0.435 and lay within the examiner-specific Boundary range.

Descriptor	E1	E2	E3	E4	E5	E6	Full range	Median	n rank 1
Boundary	0.425	0.484	0.458	0.379	0.267	0.314	0.267–0.484	0.402	6
Contour	0.083	0.021	0.275	0.131	0.089	0.244	0.021–0.275	0.110	0
Echogenicity	0.349	0.335	0.249	0.184	0.126	0.180	0.126–0.349	0.217	0
Texture	0.229	0.178	0.210	0.095	0.122	0.252	0.095–0.252	0.194	0
Size	0.162	0.153	0.113	0.098	0.173	0.051	0.051–0.173	0.133	0
Vascularization	0.096	0.098	0.040	0.128	0.087	0.142	0.040–0.142	0.097	0
Acoustic Features	0.004	0.142	0.201	0.140	0.056	0.266	0.004–0.266	0.141	0
Number of Tumors	0.001	0.022	0.096	0.067	0.013	0.021	0.001–0.096	0.021	0

Supplementary Table S13. Central effect estimates with case-bootstrap 95% confidence intervals

The case was the resampling unit; all within-case ratings and variables were retained together. A common set of 5000 NumPy 2.3.5 Generator/PCG64 resamples (seed 42) was generated in Python 3.12.13, with two-sided percentile intervals and linear quantiles. These intervals quantify uncertainty within the same retrospective cohort and do not address selection bias, spectrum restriction, independent replication, or external validation. Two-sided permutation p-values based on 100,000 case-label permutations using average ranks and NumPy's Generator with the PCG64 bit generator and seed 42 were <0.001 for boundary disagreement versus decision disagreement, 0.002 for overall descriptor disagreement versus decision disagreement, and 0.028 for overall descriptor disagreement versus error burden.

Effect	Measure	Estimate	95% CI	Valid/total
Tumor history vs histopathology	Cramér's V	0.476	0.320–0.628	5000/5000
Boundary A0 vs histopathology	Cramér's V	0.435	0.274–0.588	5000/5000
Boundary disagreement vs decision disagreement	Spearman ρ	0.361	0.206–0.500	5000/5000
Overall descriptor disagreement vs decision disagreement	Spearman ρ	0.251	0.097–0.392	5000/5000
Overall descriptor disagreement vs error burden	Spearman ρ	0.179	0.022–0.323	5000/5000
